# Leveraging Bridge Scour Detection by Multi-Model Machine Learning and Drive-By Data

**DOI:** 10.3390/s26206612

**Published:** 2026-10-19

**Authors:** Sinem Tola, Joaquim Tinoco, Eugene J. OBrien, José C. Matos

**Affiliations:** 1 Department of Civil Engineering, University of Minho, ISISE, 4800-058 Guimarães, Portugal; jtinoco@civil.uminho.pt (J.T.); jmatos@civil.uminho.pt (J.C.M.); 2 School of Civil Engineering, University College Dublin, D04 V1W8 Dublin, Ireland; eugene.obrien@ucd.ie

**Keywords:** structural health monitoring, railway bridge, drive-by, artificial neural networks, random forest, support vector machines

## Abstract

**Highlights:**

**What are the main findings?**

- Random Forest achieved the highest overall accuracy, while ANN achieved the highest balanced accuracy in the rear-mounted 7-crossing case.
- Batch-size effects were non-monotonic: short crossing histories improved some configurations, whereas longer windows generally reduced healthy-class recognition.

**What are the implications of the main findings?**

- Combining DWT- and ARX-derived Markov descriptors with drive-by track-profile measurements provides a promising basis for bridge-level scour condition screening using train-mounted sensing.
- The results support further development towards field applications, but validation with measurements from bridges under independently confirmed scour conditions remains necessary.

**Abstract:**

This study provides a detailed computational investigation of railway bridge scour detection using multiple machine learning algorithms and drive-by sensor data. A simulation-based framework generates synthetic train crossings representing track profiles acquired by Rail Infrastructure Alignment Acquisition, a far-from-axle sensing tool installed on in-service trains. Artificial Neural Networks (ANNs), Random Forests (RFs), and Support Vector Machines (SVMs) are trained, tested, and compared for binary bridge-level classification of imposed support-stiffness-reduction conditions using features derived from the Discrete Wavelet Transform and ARX-derived impulse-response Markov parameters, under leakage-free blocked nested cross-validation. RF achieved the highest overall accuracy, while ANN achieved the highest balanced accuracy in the rear-mounted 7-crossing case. Batch-size effects were non-monotonic, with short windows improving some configurations but longer windows reducing healthy-class recognition. Sensor orientation affected classifier performance and feature sensitivity, with neither configuration consistently superior across classifiers and batch sizes. The framework is further examined through class-balancing, feature-importance, and feature-family ablation analyses. The results demonstrate numerical feasibility in the adopted simulation setting and may support future bridge-condition screening after field validation.

## 1. Introduction

Bridge scour remains difficult to detect because critical components are often submerged and inaccessible during extreme hydraulic events. Recent UK flood events and bridge failures have highlighted the need for monitoring methods to detect scour-related structural changes before visible damage or collapse occurs [1,2,3]. These structural damages illustrate the importance of scour monitoring.

Scour monitoring can be performed using direct underwater or embedded sensors, such as microelectromechanical systems-based devices and sonar, or indirectly by tracking changes in structural response [4,5]. Recent studies have also demonstrated the use of embedded and surface-bonded fibre-optic sensors for monitoring structural response under impact loading [6]. Vibration-based studies have used modal frequencies, eigenfrequencies, mode shapes, and vehicle-borne measurements to detect scour-induced stiffness loss [7,8,9,10,11]. Drive-by monitoring is particularly attractive because it reduces the need for permanent bridge instrumentation. Previous railway studies have shown that train-borne accelerations or displacement profiles can detect scour-induced stiffness reductions. However, robustness to operational variability and wider bridge conditions remains a key limitation [12,13,14].

Researchers extract damage-sensitive features from monitoring data using various methods. Wavelet-based features are widely used for damage-sensitive signal analysis because they capture local changes in both scale and position. The Continuous Wavelet Transform (CWT) has been applied to scour detection using vehicle-mounted acceleration signals, but it may be computationally expensive for large datasets [15,16]. Discrete Wavelet Transform (DWT) provides a compact, non-redundant representation and has been used successfully in structural damage detection with acceleration and bridge-response data [17,18,19,20]. This makes it suitable for extracting local and span-scale changes from Rail Infrastructure Alignment Acquisition (RILA) track-profile measurements.

System-identification features offer another way to describe damage-sensitive changes in structural response. Autoregressive (AR) and autoregressive-with-exogenous-input (ARX) models represent the relation between current and past signal values and have been used for bridge damage detection under operational variability [21,22,23,24]. In this study, ARX-derived Markov parameters are used because they provide a fixed-length impulse-response description of the bridge–track system. This avoids the difficulty of using ARX coefficients directly when model order varies across simulations and bridges.

Structural vibration data are often affected by incompleteness, noise, and operational variability. Machine learning (ML) can help address missing features from faulty sensors [24], manage high-dimensional data [25], and capture nonlinear relationships between vehicle-borne signals and scour-related structural changes [26,27]. Souza et al. [26] used drive-by acceleration features with a sparse autoencoder trained on healthy bridge responses, detecting damage from the reconstruction error between the model output and the measured signal. Although effective across varying train speeds, the method depends on a healthy baseline, excludes environmental effects, and was tested with a single vehicle type and sensor orientation. Hajializadeh [27] converted simulated train-borne bogie accelerations into CWT time–frequency maps and used them as inputs to a 2D Convolutional Neural Network (CNN) for damage classification. However, the study remained a numerical feasibility test for a single bridge–train configuration and did not account for environmental effects.

Recent ML studies have also targeted scour damage more directly. Fernandes et al. [28] used an unsupervised deep autoencoder for binary scour anomaly detection in multi-axle railway bridge simulations with different sensor placements, while Gasparotti et al. [29] applied a supervised CNN to classify scour severity in a highway bridge model with a single-degree-of-freedom vehicle and fixed sensor location. Both studies represented minor and significant scour through 5% and 10% reductions in pier-foundation stiffness [28,29]. Despite these advances, ML-based vibration studies for scour detection remain limited compared with studies on other damage types or scour-depth prediction. Existing work remains constrained by sensitivity to operational and environmental variability [28,29], fixed or limited sensor placement [29], and simplified scour scenarios, often involving a single scoured pier [28,29]. These limitations motivate the use of broader simulated crossing datasets, structural dynamics, and bridge–vehicle interaction models to improve robustness, support generalisation, and compensate for the lack of field data from known scoured states [26,27,28,29].

The performance and generalisation capability of ML models depend strongly on feature selection and hyperparameter calibration. Feature selection reduces dimensionality, improves accuracy, and increases computational efficiency by removing irrelevant or redundant inputs, commonly through filter, wrapper, or embedded approaches [30]. Interpretable methods, such as SHapley Additive exPlanations (SHAP), have also been used to assess feature importance, although they can be computationally demanding [31]. For drive-by bridge anomaly detection, Corbally et al. [32] used a knowledge-transfer wrapper framework with the Binary Slime Mould Algorithm to select damage-sensitive features from vehicle-mounted accelerations. They obtained the features from a calibrated finite element model, validated them with healthy-state bridge measurements, and transferred them to real-bridge monitoring using a one-class Support Vector Machine (SVM). Although detection accuracy was high, experimental severity distinction remained challenging. Recent work has also explored multi-view feature fusion for drive-by monitoring, combining nonlinear embeddings from multiple sensor channels into a compact representation for structural-state identification [33]. Hyperparameter calibration also affects model accuracy and generalisation. Previous drive-by monitoring studies have used grid search, random search, and Bayesian optimisation to tune ML models [27,34]. However, transferability across bridge types, geometries, and operating conditions remains insufficiently verified.

Previous work by Tola et al. [13] demonstrated the viability of using train-mounted multi-sensor measurements for bridge scour detection. That study validated Fitzgerald et al. [12], in which scour is represented by reducing the vertical stiffness of the support, using both synthetic simulations and field measurements. It used drive-by displacement data rather than accelerations, with RILA measurements collected from in-service trains. Healthy-stage track profiles were obtained from an existing railway bridge, while scoured-stage profiles were synthetically generated because field data from known scoured states were unavailable. The difference between the optimised pier stiffnesses in the healthy and scoured stages was then used as the scour indicator.

More recently, Tola and Tinoco [35] presented a data-driven bridge scour monitoring study combining a taxonomy of ML-based approaches with a simulation-based railway drive-by case study. The case study introduced the overall framework for bridge-level scour detection and support-level localisation using longitudinal track-profile measurements, DWT- and ARX-derived Markov descriptors, multiple classifiers, and batch-based decisions. The present study focuses specifically on the bridge-level detection component of the framework presented earlier [35] and provides a detailed computational treatment of its feature formulation, classifier behaviour, data balancing, feature importance, sensor-orientation effects, and Markov-order sensitivity.

Against this background, the present study provides a focused computational investigation of the bridge-level detection component of the previously reported drive-by framework [35]. Using the same underlying simulation setting, the study examines in greater detail how scour-sensitive track-profile descriptors, classifier behaviour, data balancing, sensor orientation, and repeated-crossing information affect binary bridge-level detection. The specific novel contributions are as follows:

- Application of the DWT- and ARX-derived Markov descriptors to track profiles for scour detection;
- A consistent comparison of Artificial Neural Network (ANN), Random Forest (RF), and SVM classifiers using the same feature space and validation procedure;
- An assessment of the effect of Synthetic Minority Oversampling Technique (SMOTE) balancing through comparison with naturally imbalanced RF models;
- Model-specific feature-importance analysis to examine how the three classifier families use the available descriptor space;
- Evaluation of scour detection performance across different imposed support-stiffness-reduction intervals;
- Markov-order sensitivity analysis for the front- and rear-mounted sensing configurations and different crossing-batch sizes.

## 2. Methodology

**Figure 1:**
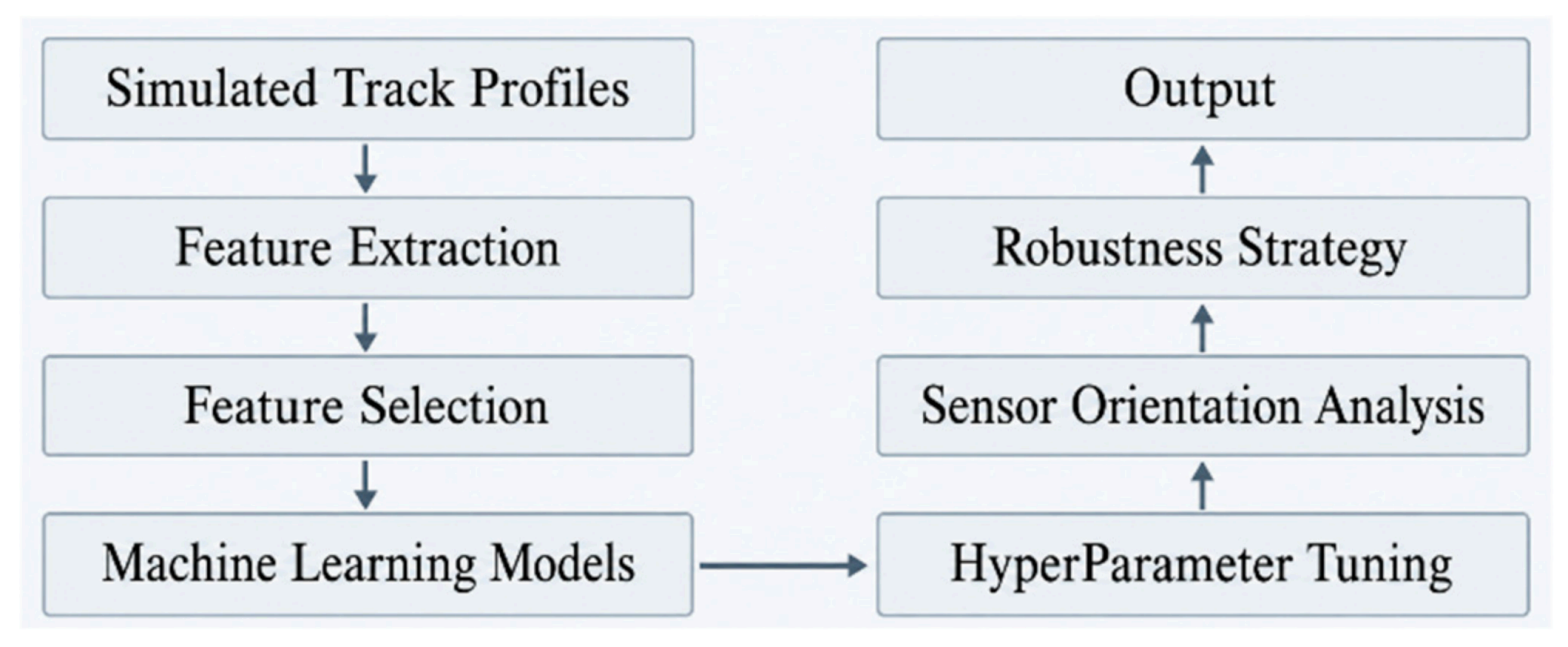
Workflow for the bridge-level detection analysis.

**Figure 2:**
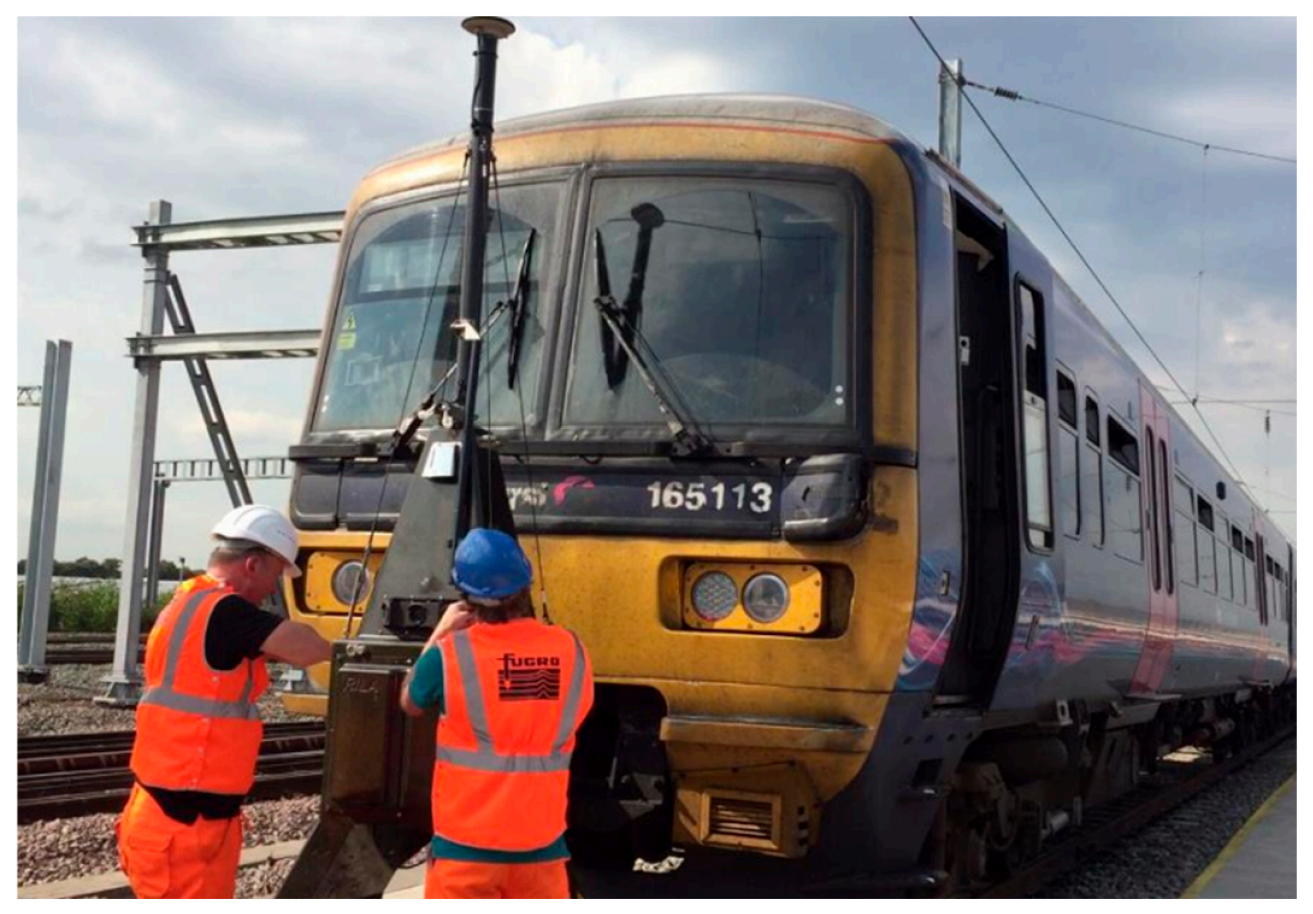
RILA implemented on a train.

The computational workflow examined in this study is illustrated in Figure 1. Its broader drive-by monitoring framework has been reported previously in Tola and Tinoco [35]. This study focuses on bridge-level scour detection and documents its computational implementation and sensitivity analyses in greater detail. Simulated track profiles are generated with TTB-2D v4.4 [36] for different train configurations, bridge types, and scour scenarios. These far-from-axle profiles, represented by Figure 2, are used for feature extraction. Two feature sets are derived: DWT coefficients, which capture scour-sensitive signal components, and ARX-based Markov parameters, obtained from impulse responses calculated using ARX coefficients. Feature selection uses relative-importance ranking and Non-dominated Sorting Genetic Algorithm II (NSGA-II). The selected features are used to train and evaluate ANN, RF, and SVM models in MATLAB 2026a. ANN hyperparameters are optimised by grid search, while RF and SVM use random search. The study then compares front- and rear-mounted RILA configurations. Finally, a sliding-window batch strategy combines consecutive crossings to evaluate how recent crossing history affects classification performance.

### 2.1. Drive-By Monitoring Concept

Drive-by displacements are measured using the RILA system of Figure 2 [37]. More details are given by Tola et al. [13]. Displacement measurements provide smoother signals than acceleration measurements; therefore, they suit ML feature extraction. However, field measurements are often very scarce. Since developing robust ML models requires large amounts of data, the present framework employs Cantero’s Vehicle–Bridge Interaction (VBI) model to generate synthetic RILA-type track profiles.

### 2.2. Simulation Framework

*Sensor orientation*: Two sensor configurations model the RILA front- and rear-mounted cases. The front-mounted sensor is placed 2 m ahead of the first axle, while the rear-mounted sensor is placed 2 m behind the last axle. The front-mounted configuration records the profile as the train first engages the bridge, capturing more immediate local responses near affected supports. The rear-mounted configuration records the response after successive bogies have crossed, allowing it to capture more accumulated global dynamics and longer-wavelength effects. This distinction is consistent with Berggren [38], who reported different behaviour between front- and rear-axle rolling-stiffness measurements. Modelling both orientations lets the framework assess sensor-placement effects under realistic RILA configurations.

*System model*: Track profiles induced by railway vehicles are calculated using TTB-2D v4.4 [36], which includes both rail irregularities and bridge displacements [13]. The model represents the bridge using Euler–Bernoulli beam finite elements with elastic/viscous support conditions, the track through rail–pad–sleeper–ballast components, and the train as a multibody vehicle model with primary and secondary suspensions. The vehicle, track, and bridge subsystems are coupled through wheel–rail contact, and the coupled equations are integrated using the Newmark-β method.

RILA is a spatially referenced mobile measurement system. Its sensors acquire data at fixed temporal sampling rates, while the measurements are associated with positions along the track using the system trajectory, with GNSS and IMU information supporting spatial referencing [39]. The 0.2 m spacing adopted in the present simulations was selected based on the field RILA measurements used in our previous study [13]. Across the 16 field runs considered there, the mean consecutive spatial interval was approximately 0.203 m, with a standard deviation of 0.058 m and a median of 0.199 m. We therefore adopted 0.2 m as a representative fixed spatial spacing for the simulated RILA-type profiles. This ensures that profiles generated at different train speeds have the same spatial locations and dimensions for subsequent feature extraction. Train speed still affects the simulated vehicle–bridge interaction response, but the response is represented at fixed 0.2 m positions along the bridge. The rail-irregularity profile, generated at a finer spatial resolution, is interpolated to these same positions before being combined with the simulated bridge displacement.

For each of the four simulated bridges, one distinct Federal Railroad Administration Class 6 rail-irregularity profile is generated and retained for all crossings of that bridge. Thus, rail irregularity varies between bridges but is assumed to remain unchanged between crossings of the same bridge. Vehicle speeds range from 10 m/s to 44.7 m/s, based on UK field measurements and values reported by Wang et al. [39]. For each crossing, a Monte Carlo simulation randomly generates train speed and body weight, with body weight limited to ±10% of the technical specifications. Vehicle mechanical properties are kept constant. Three vehicle types are simulated in equal numbers: the Class 66/0 Co-Co Diesel locomotive, the Eurostar trainset, and the AVE S103 ICE3 trainset. Supplementary Table S1 lists their properties.

*Bridge Models*: The dataset contains one simulated RILA-equipped vehicle crossing for each calendar day of 2015–2016 at each of the four bridges, giving 731 crossings per bridge and 2924 crossings in total. All 2924 generated crossings are retained in the baseline dataset. It includes two concrete bridges in Northern England, WCM15 and WCM15A in Carlisle, and two steel bridges in Southern England, the River Teign near Newton Abbot and the Colonnade in Dawlish. These bridges provide structural variability in superstructure type, number and length of spans, stiffness, damping, and support conditions. Their properties are summarised in Table 1. Pier spring stiffness values are calculated using the FEMA 356 formulation, and the railway-track properties are adopted from previous work [13].

**Figure 3:**
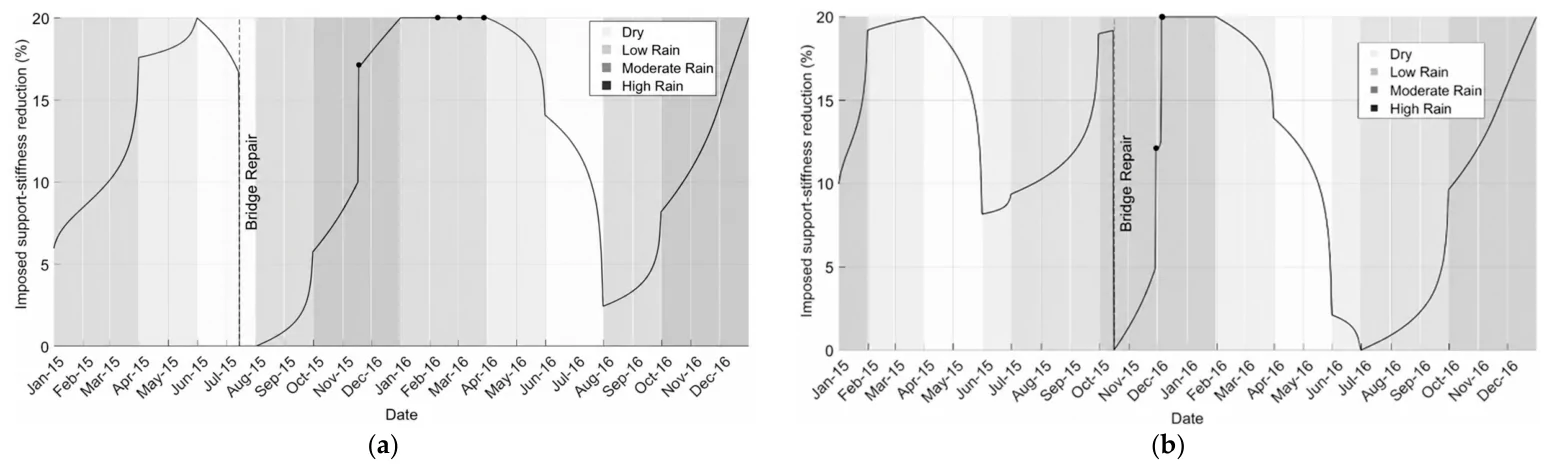
Prescribed support-stiffness-reduction histories for 2015–2016: (**a**) south; (**b**) north.

*Chronological condition sequences*: Two synthetic chronological condition sequences covering 2015 and 2016 are used for Southern and Northern England, respectively: one for Southern England (Figure 3a) and one for Northern England (Figure 3b), aligned with the respective bridge locations. These sequences are generated because the batch-based sliding-window strategy requires ordered sequences of train crossings. One crossing is simulated per day; hence, one imposed support-stiffness-reduction value is assigned to each day. These condition sequences are not products of hydraulic scour models. Rainfall categories and historical storm periods are used only to introduce temporal variation into the synthetic condition histories. The years 2015–2016 were selected because they included an unusually wet winter with major UK storm events, including Storms Desmond, Eva, Frank, Imogen, and Katie [1].

Rainfall categories are defined using 30-year precipitation records from 1991 to 2020 at the nearest UK Meteorological Office stations, Teignmouth for the south bridges and Carlisle for the north bridges [40]. A baseline imposed support-stiffness-reduction level is assigned by rainfall category: 10% for high-rainfall months (Oct–Dec); 6% for moderate-rainfall months (Jan–March and Aug–Sep); and 3% for low-rainfall months (April–July). Region-specific dry months also use a 3% base scour, with additional regression. In Figure 3, rainfall intensity is shown by background shading, from dry to high-rainfall months. These categories define the baseline support-stiffness-reduction levels, while historical storms and flood records introduce discrete scour peaks up to 20%.

The north sequence includes Storms Clodagh and Desmond, while the south sequence includes Storms Barney, Imogen, Jake, and Katie. The storm dates are marked with dots. Around each flood, scour increases linearly up to the flood day and then decreases over a ±5-day window to represent temporary increases and subsequent decreases in the imposed stiffness-reduction level. On the flood day, an additional stochastic spike is sampled from a uniform distribution, since no evidence was available to assume that flood-induced increments are normally distributed. The spike bounds depend on rainfall intensity: 6–10% for high-, 4.5–7.5% for moderate-, and 3–5% for low- or dry-rainfall months.

Between floods, seasonal transitions are modelled using a logarithmic curve between the start and end of each season, with imposed support-stiffness reduction at time *t* expressed as in Equation (1):

*y*(*t*) = *alog*(*t*) + *b* (1)

Coefficients *a* and *b* are derived as in Equation (2):

(2) $$a=\frac{y_{2}-y_{1}}{\log\left(t_{2}\right)-\log\left(t_{1}\right)},\;\;b=y_{1}-a\log\left(t_{1}\right).$$

where *y*(*t*) is the imposed support-stiffness-reduction value at day *t*; *t*_1_ and *t*_2_ are the start and end day indices of the seasonal transition, respectively; and *y*_1_ and *y*_2_ are the corresponding imposed support-stiffness-reduction values. Here, t is the positive day index within the corresponding seasonal transition, with *t*_1_ ≥ 1 and *t*_2_ > *t*_1_, so all log arguments remain positive. The logarithmic trend of scour progression is supported by formulations in the literature (Ettema et al. [41], Oliveto and Hager [42], and Kothyari et al. [43]) which describe an initial rapid increase in local scour depth, followed by slower development as equilibrium is approached.

Daily changes in the imposed support-stiffness-reduction level are set at +0.06%, +0.02%, and +0.01% for high-, moderate-, and low-rainfall months, respectively. Following floods, the daily change for low-rainfall months is set to −0.02%. For dry months, it is set to −0.04%. These reductions in the imposed support-stiffness-reduction level, and cases around 0%, should not be interpreted as soil consolidation or complete restoration of the hole. Values below 5% are included to represent the healthy class, not only the ‘scoured’ stage. Threshold details of ML models are provided in Section 2.4. A three-day pause is introduced after floods to represent temporary scour-hole stabilisation, after which scour resumes through logarithmic or linear progression. Repair resets are also included, with the imposed support-stiffness reduction reset to 0% for the north bridges in October 2015 and for the south bridges in July 2015. On public holidays, the assigned support-stiffness-reduction level is held constant; the corresponding daily simulated crossing is nevertheless retained in the dataset. These rules prescribe only temporal variation in the imposed support-stiffness reduction. The rainfall categories, peaks, seasonal transitions, temporary pauses, and repair resets are not calibrated or validated hydraulic models of scour evolution, and the resulting chronological histories should not be interpreted as physical scour-depth histories.

*Scour modelling*: Scour is modelled as a proportional reduction in the vertical stiffness of the pier springs [13]. The synthetic chronological condition sequence assigns a 0–20% reduction in vertical support stiffness at the affected supports. These percentages represent imposed structural stiffness reduction associated with reduced foundation support, not physical scour depth or the hydraulic and geotechnical scour process itself. The chronological sequences provide synthetic temporal variation between healthy and scoured conditions in the absence of field measurements from known scoured states. Most assigned conditions fall within the 5–20% stiffness-reduction range, providing sufficient representation of scoured cases for classifier development. A maximum of three supports are modelled as scoured simultaneously. The first support is randomly selected, with any additional scoured supports adjacent to it, representing progressive scour development around neighbouring piers. For each simulated case, the affected support location is selected and is not constrained to remain fixed throughout a prescribed temporal event; when more than one support is affected, the additional supports are adjacent to the initially selected support.

*Noise injection*: Gaussian noise with zero mean and a fixed variance is injected into each simulated track profile, with the standard deviation defined as in Equation (3) and the noisy signal expressed as in Equation (4):

*σ* = 0.005 × *RMS*(*δ*) (3)

**Figure 4:**
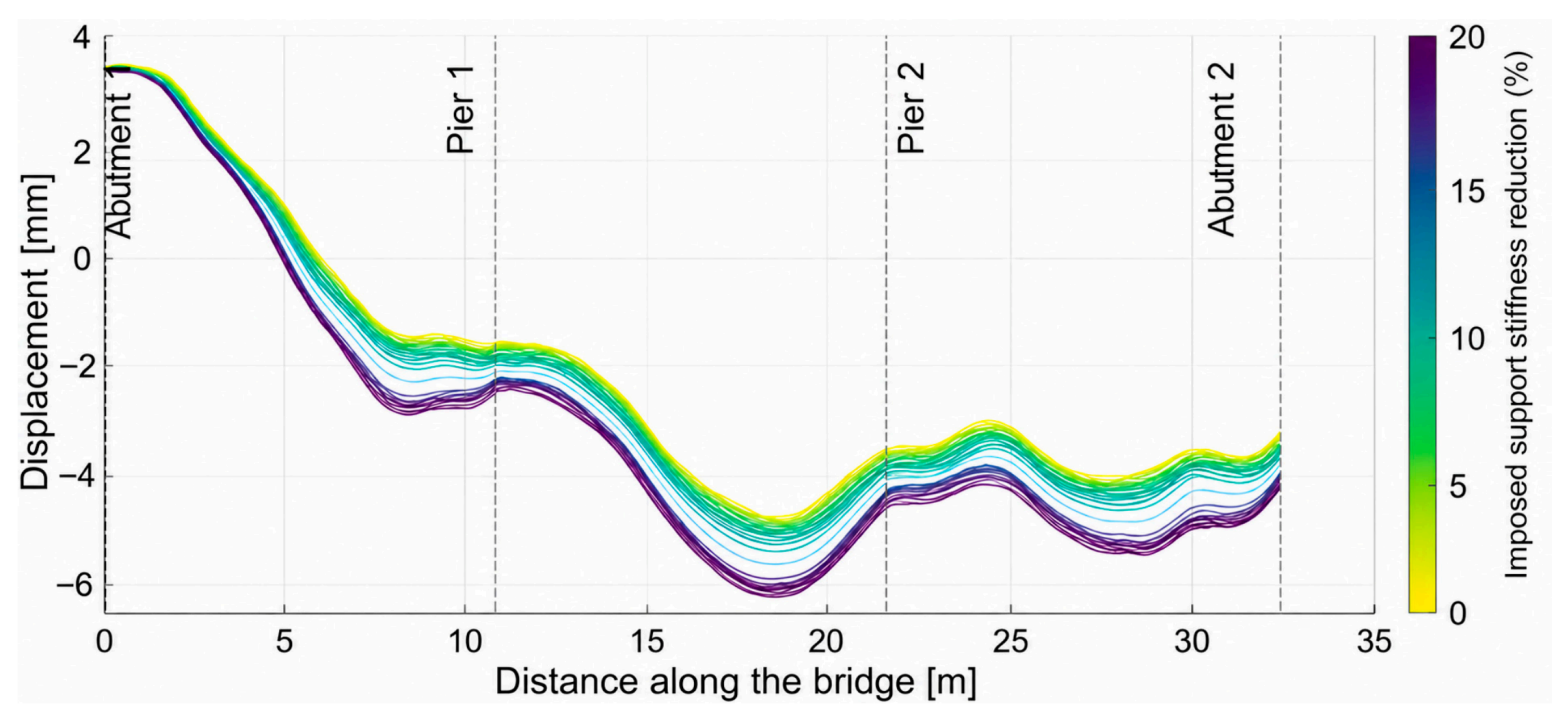
Representative simulated RILA front-mounted track profiles for the Colonnade bridge.

*δ_noisy_* = *δ* + *n*, *n*∼*N*(0, *σ*^2^) (4)

where *δ* denotes the simulated track profile, *n* is the noise, *σ* is the standard deviation of the added noise, and *RMS*(*δ*) is the root mean square of *δ*. This yields moderate noise proportional to the signal magnitude, consistent with the filtered nature of RILA field data [13]. Figure 4 shows 30 representative RILA-acquired simulated track profiles along the Colonnade bridge when the sensor is implemented in front of the vehicle. The colour bar shows the imposed support-stiffness-reduction level for each crossing. Profiles with lower stiffness-reduction levels appear in yellow–green tones and cluster closer to the baseline response, whereas higher stiffness-reduction levels appear in blue–purple tones and tend to produce larger displacement changes.

### 2.3. Signal Processing and Feature Engineering

Two complementary feature sets are derived from track profiles: (i) Type A: features derived from DWT coefficients, and (ii) Type B: Markov parameters obtained from ARX models.

*Application of DWT to track profiles*: DWT enables the detection of singularities, trends, and local variability [44], which are useful for scour detection. It provides a multi-resolution representation, yielding sets of approximation (low-frequency) and detail (high-frequency) coefficients [44,45]. The track profile is the discrete sequence as in Equation (5):

(5) $$\delta ={\left\{\delta (x_{n})\right\}}_{n=0\;}^{N-1}$$

where *N* is the number of samples in *δ*, *n* is the discrete spatial sample index, and *x_n_* is the spatial location of sample *n* on a uniform grid, *x_n_* = *x*_0_ + *n* ∙ ∆*x*. The DWT applies low-pass and high-pass filters to the track profile using half-point symmetric extension at both boundaries, thereby limiting boundary artefacts. Approximation and detail coefficients are produced, respectively, as defined in Equations (6) and (7).

(6) $$a_{1k}=\Sigma_{m=0}^{M-1}h[m]\cdot \delta^{\mathrm{sym}}(2k-m)$$

(7) $$d_{1k}=\Sigma_{m=0}^{M-1}g[m]\cdot \delta^{\mathrm{sym}}(2k-m)$$

where *k* is the wavelet coefficient index at a given level *j*; *M* is the filter length; *M* = 2*p*; *p* is the number of vanishing moments; *k* is the coefficient index: *k* = 0, 1, …, ⌈*N*/2∙*j*⌉ − 1; and *δ*_sym_ is given in Equation (8):

*δ*^sym^(*x_n_*) = {*δ*(*x*_−*n*−1_), (*n* < 0); *δ*(*x_n_*), (0 ≤ *n* ≤ *N* − 1); *δ*(*x*_2*N*−*n*−1_), (*n* ≥ *N*)} (8)

Recursively applying the first step to the approximation yields Equations (9)–(11); for *j*:

*a*_0_(*n*)= *δ*(*n*); (9)

(10) $$a_{\mathrm{jk}}=\Sigma_{m=0}^{M-1}h[m]\cdot a_{j-1}^{\mathrm{sym}}(2k-m)$$

(11) $$d_{\mathrm{jk}}=\Sigma_{m=0}^{M-1}g[m]\cdot a_{j-1}^{\mathrm{sym}}(2k-m)$$

where *j* = 1, 2, …, *J*, and *J* is the decomposition level. Reconstruction from level *j* to *j* − 1 (upsample-by-2 and filter) is given by Equation (12):

*a_j_*_−1_(*n*) = *Σ_k_*ḥ[*n* − 2*k*] · *a_jk_* + *Σ_k_ ġ*[*n* − 2*k*] · *d_jk_* (12)

where *ḥ*, *ġ* are the orthogonal cases: *ḥ* = *h*, *ġ* = *g*. The discrete analysis/synthesis filters *h*[*m*] and *g*[*m*] are generated by the continuous two-scale relations for the scaling function *ϕ* and wavelet equation *ψ*, as seen in Equations (13) and (14), respectively:

(13) $$\phi (x)=\sum_{m=0}^{M-1}h[m]\surd 2\phi (2x-m)$$

(14) $$\psi \left(x\right)=\sum_{m=0}^{M-1}g[m]\surd 2\phi (2x-m)$$

DWT therefore represents the track-profile signal as a combination of approximation (Equation (6)) and detail coefficients (Equation (7)). The orthogonal Daubechies family is adopted because it provides smooth approximation coefficients suitable for track-profile representation. The db8 wavelet and decomposition level *J* = 4 are selected because they capture scour-related changes without appreciable improvement at higher orders or levels.

**Figure 5:**
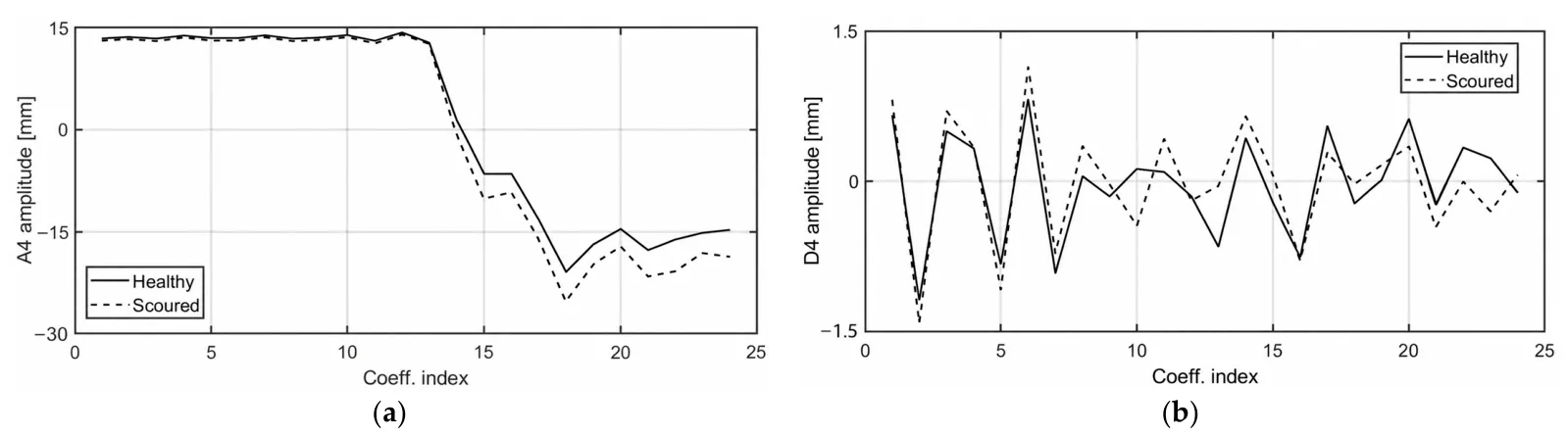
DWT (db8, level 4) of track profiles for the Colonnade bridge under healthy and ≈20% imposed support-stiffness-reduction conditions: (**a**) approximation coefficients (*A*_4_); (**b**) detail coefficients at level 4 (*D*_4_).

Figure 5 illustrates scour-related changes in the wavelet coefficients. The approximation (*A*_4_) and level 4 detail coefficients, i.e., *D*_4_, of one healthy and one scoured-stage track profile of the Colonnade bridge are shown in Figure 5a and Figure 5b, respectively. The healthy track-profile coefficients are plotted as a solid curve, while the coefficients of the track profile with an imposed support-stiffness reduction of 20% at pier 2 are shown with the dashed curve. Figure 5a shows clear differences between healthy and scoured *A*_4_ coefficients, indicating scour-induced changes in the low-frequency response. Among the detail levels, the most pronounced scour-related differences appeared at level 4, where the damaged case shows higher energy (ratio ≈ 1.25) and a larger mean absolute difference (≈0.25 mm) than the healthy profile.

*Type A (DWT-based) features*: The bridge-level DWT descriptor set follows that reported in [35] and is detailed mathematically here for completeness and reproducibility. Each crossing is decomposed with db8 to the maximum level *J* = 4, yielding the level 4 approximation vector *A*_4_ = {*a*_4_ [*k*]} and the detail vectors *D_j_* = {*d_jk_*} for *j* = 1, …, 4. Then, for all coefficient vectors *z* ∈ {*A*_4_, *D*_1_, *D*_2_, *D*_3_, *D*_4_} with length *n_z_*, a total of 12 vectors are derived. These are the skewness and kurtosis of *A*_4_ and of each *D_j_* (5 skewness + 5 kurtosis), the total wavelet-domain energy, and the minimum of *A*_4_.

- Skewness of approximation and detail coefficients *A*_4_, *D*_1_, …, *D*_4_, are given in Equation (15):

(15) $$\mathrm{Skw}\left(z\right)=\frac{1}{n_{z}}\sum_{i=1}^{n_{z}}{\left(\frac{z_{i}-\mu_{z}}{\sigma_{z}}\right)}^{3}$$

- Kurtosis of approximation and detail coefficients *A*_4_, *D*_1_, …, *D*_4_, are given in Equation (16):

(16) $$\mathrm{Krt}\left(z\right)=\frac{1}{n_{z}}\sum_{i=1}^{n_{z}}{\left(\frac{z_{i}-\mu_{z}}{\sigma_{z}}\right)}^{4}$$

- Total wavelet-domain energy, by Parseval’s theorem [46], is given in Equation (17):

(17) $$E_{\mathrm{tot}}=\sum_{i=1}^{n_{z}}A_{4}{\left(i\right)}^{2}+\sum_{j=1}^{J}\sum_{i=1}^{n_{z}}\left(D_{j}^{2}(i)\right)$$

The total wavelet-domain energy is calculated as the sum of squared coefficients across *A*_4_ and *D*_1_–*D*_4_; no additional normalisation by profile length or coefficient count is applied.

- The minimum value of the approximation coefficient is given in Equation (18):

(18) $$L_{\min}^{A}=\underset{i}{\min}A_{4}(i)$$

This feature set targets two expected scour effects in the spatial track profile: span-scale downward shifts with long-wavelength baseline distortion, and localised irregularities near the affected pier. Skewness and kurtosis describe coefficient asymmetry and sharp local changes in slope or curvature, while total wavelet energy reflects the cumulative signal energy distributed across the wavelet scales. The minimum of *A*_4_ captures the deepest long-wavelength dip, which increases with scour. *D*_1_*–D*_3_ describe pier-vicinity variations, whereas *D*_4_ and *A*_4_ capture longer-wavelength, span-scale changes.

The contribution of the Type A features is assessed using the relative-importance (RI) analysis of Cortez and Embrechts [47]. The method evaluates the dispersion of sensitivity responses *y_a_* to each input feature *x_a_*. The variance *S_v_* in Equation (19) is used since it is statistically interpretable and less sensitive to outliers.

(19) $$S_{v}=\frac{\sum_{j=1}^{L}{\left(y_{a}^{j}-\mathrm{mean}\left(y_{a}\right)\right)}^{2}}{L-1}$$

The relative importance of each parameter, *r_a_*, is defined in Equation (20):

(20) $$r_{a}=\frac{M_{a}}{\sum_{i=1}^{M}M_{i}}$$

where *j* = 1, 2, …*L*; *L* is the number of sensitivity evaluations carried out for each input feature, chosen as *L* = 100; *i* = 1, 2, …, *M*, where *M* denotes the total number of parameters; and *M_a_* = *S_v_* is the sensitivity measure chosen for feature *a*.

This analysis showed that one or two Type A features dominated the importance scores in ML models. This raised the risk of fitting dataset-specific correlations rather than learning the scour-sensitive behaviour. Therefore, Type B features were introduced using system-identification models of track-profile dynamics.

*Type B (ARX-based) features*: The bridge-level ARX/Markov formulation follows that reported in [35] and is detailed here to provide the complete computational formulation used in the present detection analysis. The observed track profile along the bridge is given by Equation (21):

*δ*(*x*) = *ξ*(*x*) + *z*(*x*) (21)

It comprises the rail irregularity segment *ξ*(*x*) and the bridge displacement response *z*(*x*). The rail irregularity is present in the output at the same spatial location. For each simulation, rail irregularity is used as the exogenous input and the track profile as the output. The adequacy of this input–output formulation is assessed through residual autocorrelation and input–residual cross-correlation diagnostics.

In the implemented ARX identification procedure, the rail-irregularity input was manually shifted by one spatial sample before fitting the model. Denoting the original spatially aligned rail irregularity by *ξ*(*n*), the input supplied to the ARX identification was *v*(*n*) = *ξ*(*n* − 1), with zero padding at the first sample. The ARX model was subsequently estimated with an additional specified input delay of *n_k_* = 1. Thus, the combined implementation introduces an effective delay of two spatial samples (0.4 m) relative to the original rail-irregularity sequence. The transfer-function and Markov-parameter definitions in Equations (22)–(27) refer to the shifted input *v*(*n*). This delay represents the adopted identification convention and should not be interpreted as evidence of a physical absence of instantaneous rail-irregularity effects. With sampling step *T_s_* = 0.2^m^, the ARX model is formulated in Equation (22):

*A*(*q*^−1^)*δ*(*n*) = *B*(*q*^−1^)*q*^−*nk*^*v*(*n*) + *e*(*n*) (22)

(23) $$A(q^{-1})=1+a_{1}\;q^{-1}+a_{2}\;q^{-2}+\cdots +a_{\mathrm{na}}\;q^{-n_{a}}$$

(24) $$B(q^{-1})=b_{1}+b_{2}\;q^{-1}+\cdots +b_{n_{b}}\;q^{-(n_{b}-1)}\;$$

where *a_i_* and *b_i_* are the autoregressive and input coefficients, respectively, and *q*^−1^ is the backward shift operator defined as *q*^−1^*δ*(*n*) *= δ*(*n* − 1). The term *A*(*q*^−1^) in Equation (23) is the autoregressive polynomial that encodes the dependence of output δ(n) on its past values. *B*(*q*^−1^) in Equation (24) is the input polynomial, representing the contribution of previous input samples; *n_a_* and *n_b_* are the autoregressive and input orders, respectively; *n_k_* = 1 is the fixed input delay (one spatial step); and *e*(*n*) denotes the residual.

ARX model orders are selected separately for each simulation by setting *n_a_* = *n_b_* and minimising the Akaike Information Criterion (AIC). AIC compares competing models by balancing goodness of fit and model complexity. Lower AIC values indicate the preferred model [48]. For WCM15, WCM15A, and River Teign, candidate orders from 1 to 60 are evaluated; their bridge-profile segments contain 482, 598, and 464 spatial samples, respectively. The Colonnade is considerably shorter, with 162 spatial samples over approximately 32.4 m. To avoid an excessive model order relative to the available spatial observations and the unstable high-order fits identified during model diagnostics, the Colonnade search is limited to orders 1–30, and the minimum-AIC model is selected among stable candidates. Thus, the maximum Colonnade model contains 30 autoregressive and 30 input coefficients for 162 spatial observations. The same order-selection procedure was applied independently to the front- and rear-mounted Colonnade profiles.

The selected ARX orders varied across bridge and sensor configurations; Supplementary Table S7a reports the full distributions. For the leading configuration, the median (Q1–Q3) orders were 10 (9–12) for WCM15, 8 (8–10) for WCM15A, 8 (7–8) for River Teign, and 16 (7–24) for Colonnade. For the trailing configuration, the corresponding values were 11 (9–14), 9 (8–10), 8 (7–9), and 6 (5–9), respectively. For Colonnade, the selected-order ranges were 5–30 for leading and 4–30 for trailing, and all selected models were stable.

For the six configurations using the standard 1–60 ARX search, the direct-feedthrough formulation produced no systematic improvement in AIC: the median Δ*AIC* = *AIC_nk=_*_0_ *− AIC_current_* ranged from −0.0064 to +0.0014. Stability ranged from 98.36% to 100% for the current formulation and from 98.50% to 100% for the direct-feedthrough formulation. Residual-autocorrelation pass rates were 69.25–91.99% and 69.54–92.13%, respectively, whereas the input–residual cross-correlation diagnostic passed the approximate 99% confidence bounds for 100% of the evaluated models in all six configurations under both formulations. Thus, some models retain residual serial correlation, indicating remaining unmodelled dynamics, but the input–residual diagnostics do not indicate a systematic dependence between the exogenous input and identification residuals. The ARX models are therefore used here primarily as compact feature generators rather than as complete stochastic descriptions of the bridge–track dynamics.

In the original implementation, the rail-irregularity sequence was shifted by one spatial sample before ARX identification, and the ARX structure was specified with *n_k_* = 1. This identification convention was used to obtain a delayed input–output representation and is not interpreted as evidence that the physical system has no instantaneous response. To assess sensitivity to this choice, a targeted diagnostic comparison was performed at the same selected ARX order using (i) the original shifted-input *n_k_* = 1 formulation, (ii) the spatially aligned input with *n_k_* = 1, and (iii) the spatially aligned input with *n_k_* = 0, which permits direct feedthrough. These diagnostic calculations were performed only to verify the model and did not regenerate the Markov features or alter the subsequent ML models.

Parametric ARX identification was successful for most simulations. For the RILA trailing River Teign configuration, ARX identification succeeded for 348 of the 731 simulations. The remaining 383 cases were handled using KNN-based coefficient completion. Similarity between simulations was assessed using three signal-derived descriptors: the standard deviation and root mean square of the detrended track profile, and its correlation with the detrended rail-irregularity input. These descriptors were standardised using statistics calculated from successful ARX fits, and Euclidean distances were used to identify the five nearest successful simulations.

Because successfully identified ARX models had different polynomial orders, their coefficient vectors were truncated to common lengths determined from the minimum available lengths among successful models. The leading unity coefficient of the autoregressive polynomial was excluded during coefficient averaging and reinstated after imputation. Each missing coefficient vector was estimated using a weighted combination of the component-wise mean of the five nearest successful models (*α* = 0.7) and the component-wise median of the successful-model population (1 − *α* = 0.3). The estimates were subsequently clipped within a median ±6 median-absolute-deviation (MAD) envelope, and a small deterministic perturbation was applied to avoid identical imputed coefficient vectors.

The imputed cases were retained in the ML dataset but excluded from ARX-specific order, stability, residual, and direct-feedthrough diagnostics. For held-out cases, the imputation donors and associated statistics, including means, standard deviations, medians, and median absolute deviations, were derived exclusively from the corresponding training partitions, without using the true damage labels of held-out cases.

For simulations with successful parametric identification, the ARX polynomial orders varied between cases, making the raw ARX coefficients unsuitable as a fixed-dimensional ML input. To obtain the same number of ML model input features across simulations, the ARX models are re-expressed in terms of their Markov parameters, i.e., the impulse-response coefficients [49], using the transfer-function formulation in Equation (25):

(25) $$G\left(q^{-1}\right)=\frac{q^{-\mathrm{nk}}\;B\left(q^{-1}\right)}{A\left(q^{-1}\right)}=\sum_{k=0}^{\infty }g\left(k\right)q^{-k}$$

The Markov parameters are defined recursively as in Equation (26):

(26) $$g\left(k\right)=\tilde{b}\left(k\right)-\sum_{i=1}^{\min\left(n_{a},\;k-1\right)}a_{i}\cdot g\left(k-i\right),k=1,2,\ldots H-1$$

where *k* is the impulse index; the delay-aligned input coefficient $\tilde{b}\left(k\right)$ is defined as $\tilde{b}\left(k\right)$ *= b_k−nk+_*_1_ if 1 ≤ *k* − *n_k_* + 1 ≤ *n_b_*, and zero otherwise. In this study, *g*(*k*) represents the impulse-response coefficient at spatial lag *k*. A fixed reference horizon of *H* = 40 spatial samples, corresponding to 8 m at a sampling interval of 0.2 m, was adopted for the Markov representation. The cumulative impulse-response energy was evaluated within this reference window, and the proportion accumulated by each shorter horizon was computed relative to the energy of the 40-sample response. For each simulation with finite, non-negligible reference-window energy, the shortest horizon reaching 95% of this reference-window energy was determined. Accordingly, this assessment characterises the distribution of energy within the selected 40-sample representation and does not establish the fraction of the infinite-horizon response energy captured by *H* = 40. For WCM15, WCM15A, and River Teign, stability was assessed diagnostically rather than imposed as a model-selection constraint; finite impulse responses from the selected ARX models were retained in the reference-window energy assessment, including those from unstable fits. For Colonnade, only stable ARX candidates were eligible for selection.

Finite impulse responses obtained from the identified ARX models or, where needed, from non-parametric impulse-response estimation, regularised FIR fallback, and River Teign KNN completion were included in the reference-window energy assessment. Imputed ARX coefficients were not treated as successfully identified models in the separate ARX order, stability, and residual diagnostics. The resulting feature vector per simulation is seen in Equation (27):

*g* = [*g*(1), *g*(2), …, *g*(39)]*^T^* (27)

which provides a uniform description of the bridge–track dynamics. Unlike ARX coefficients, which vary in number and scale with model order, Markov parameters provide a fixed-length, order-invariant description of the system dynamics. MATLAB uses one-based array indexing; therefore, the 40 stored impulse-response samples occupy computational positions 1–40 but correspond mathematically to *g*(0), …*g*(39). With the adopted *n_k_* = 1 formulation, *g*(0) is structurally zero and is excluded. The ML feature set therefore contains the 39 Markov descriptors *g*(1), …*g*(39), which, together with the 12 DWT descriptors, give 51 features.

### 2.4. Modelling Approach

*Binary classification*: Simulations with an imposed vertical support-stiffness reduction of 5% or greater are labelled as scoured, while those below 5% are labelled as healthy. The 5% value is an adopted decision boundary for the present simulation-based classification problem, rather than a universal physical distinction between healthy and scoured foundations. It was selected because previous drive-by scour studies used 5% as the lowest simulated condition [28,29], while earlier sensitivity analysis within the adopted modelling framework showed track-profile deviations from this lower range that increased with stiffness loss [13]. The threshold therefore defines the two classes used for ML evaluation and should not be interpreted as a threshold in physical scour depth or severity. Class-specific metrics are interpreted accordingly: the F1 score for the scoured class (5–20%) measures detection of scoured cases, while the F1 score for the healthy class (<5%) measures recognition of healthy cases and therefore relates to false-alarm reduction. The threshold was specified a priori and was not optimised using outer-test performance.

*Feature Selection*: With the adopted *n_k_* = 1 formulation, the 40-sample impulse-response vector is indexed as *g*(0), …*g*(39). The zero-lag coefficient *g*(0) is structurally zero because of the imposed one-sample input delay and is therefore excluded from the ML feature set. The retained Markov descriptors are consequently *g*(1), …*g*(39), giving 39 Markov features and 51 features in total when combined with the 12 DWT descriptors. The zero value of *g*(0) results from the adopted model structure and is not interpreted as evidence of no instantaneous physical response. Alternative feature subsets are evaluated separately through the feature-selection analyses described below, but the final classifier comparison retains the full 51-feature space. This ensures comparability between algorithms and reproducibility. Two feature selection strategies are applied:

i. *Relative-Importance-Based Selection with Grid Search*: Feature importances are estimated through perturbation-based sensitivity analysis. The variance of model outputs with respect to random perturbations of each feature is used to compute relative-importance scores (Equation (20)). Features are then ranked in descending order of importance. Using this ranking, subsets of increasing size are formed from the top 1 feature to the complete set of 51 features. Then, each subset is evaluated through external 5-fold cross-validation (CV) of the ANN classifier. The selection criteria prioritised the F1 score for the scoured class (5–20%), as scour detection requires minimising false negatives (safety-driven). A hierarchical decision rule is applied:
  1. Require an F1 score for the scoured class (5–20%) ≥ 0.85 (safety floor).
  2. If multiple subsets satisfy this, prefer the largest number of features.
  3. In case of ties, maximise MacroF1, balanced accuracy, and Matthews Correlation Coefficient (MCC), in that order.
ii. *Multi-Objective Feature Selection using NSGA-II*: NSGA-II [50] is applied as a wrapper feature-selection method with two objectives: maximising the F1 score for the 5–20% stiffness-reduction class and minimising the fraction of selected features. The algorithm uses a population size of 100, a crossover fraction of 0.8, and 40–60 generations, depending on convergence. Parallel evaluation is used for population fitness calculations, and a Pareto spread tolerance of 10^−3^ defines convergence. From the Pareto front, the subset with the largest number of features satisfying the F1 score for the scoured class (5–20%) ≥ 0.85 is selected.

*Assessment and Decision*: The feature-selection results are given in Table 2. Both approaches give similar metrics. NSGA-II shows that compact subsets can capture scour-sensitive dynamics. However, the full 51-feature set is retained for the final classifier comparison to maintain a common descriptor space and ensure cross-model comparability across ANN, RF, and SVM. Its advantage over more compact subsets is evaluated separately through feature-family and subset ablation analyses. These feature-selection analyses therefore assess alternative descriptor subsets but do not define a reduced feature set for the final classifier evaluation.

*Parameter Normalisation*: Within each CV iteration, the normalisation parameters are estimated using only the corresponding training fold. For ANN and SVM, z-score normalisation uses the training-fold mean and standard deviation, and the same parameters are then applied unchanged to the corresponding evaluation fold. For the ANN and SVM classifiers, z-score normalisation is applied; i.e., each feature is centred by subtracting its mean and scaled by dividing by its standard deviation. This step is required for ANN and radial basis function (RBF)-SVM because ANN training is gradient-based and RBF-SVM is distance-based. Without scaling, large-magnitude features could dominate weight updates or distort the kernel space. For RF, scaling is unnecessary because decision-tree splits depend on feature ordering rather than on magnitude.

*Batch-Based Scour Detection*: To reduce the influence of noisy individual crossings and improve robustness, a sliding-window strategy is applied to the feature space. A single-crossing case (*m* = 1) is also evaluated as a no-window baseline. Batch sizes of 7, 15, 31, and 61 crossings corresponding to 1 week, 2 weeks, 1 month, and 2 months are considered. Each window advances by one crossing. For a window of length *m*, the batch feature vector includes two descriptor groups. These are aggregated statistics (mean, standard deviation, minimum, and maximum) for each feature across the m crossings, plus the individual feature descriptors for each crossing, which preserve short-term temporal information. The combined input batch vector has dimension (*m* + 4)∙*f*, where *f* is the number of input features per crossing. Because this dimension depends on m, we generated the batch representation separately for each batch size, only after assigning individual crossings to the corresponding folds of the nested CV. The class of the final crossing in each window defines the batch label. Assuming one crossing per day, a batch of m crossings requires m observations to initialise; thereafter, the window advances by one crossing, and an updated classification is produced after each subsequent crossing.

This strategy represents a near-real-time monitoring setting, where previous crossings provide stabilising context, and the most recent crossing defines the decision. To avoid information leakage arising from overlapping sliding windows, data partitioning was performed at the individual-crossing level using blocked chronological folds. Within each nested-CV iteration, windows were constructed separately within the corresponding folds, and a window was retained only when all of its constituent crossings belonged to the same fold; windows spanning fold boundaries were discarded. Thus, consecutive windows may overlap within a fold, but no crossing is shared between the outer-training and outer-test folds or between the inner-training and inner-validation folds. For each sensor orientation and batch size, the same outer-fold crossing partitions were used for ANN, RF, and SVM, ensuring that all classifiers were evaluated on identical outer-CV test folds. The same window-exclusion rule was applied during inner cross-validation for hyperparameter optimisation. Effective sample counts and corresponding healthy/scoured-class counts and proportions after excluding windows spanning CV fold boundaries are reported in Supplementary Table S6. The resulting totals are 2924, 2804, 2644, 2324, and 1724 for *m* = 1, 7, 15, 31, and 61, respectively.

*Data Balancing*: The chronological condition sequences in Figure 3 are imbalanced because rainfall and flood periods dominate, resulting in fewer crossings in the healthy class (<5% imposed support-stiffness reduction). To reduce majority-class bias, SMOTE [51] is applied only within the training data of each CV split. Validation and test folds contain only original simulation samples not generated by SMOTE. During inner CV, SMOTE is applied only to the inner-training subset; after hyperparameter selection, it is applied only to the full outer-training fold.

SMOTE uses *k* = 5 nearest minority-class neighbours and deterministic random seeds implemented using MATLAB’s Twister generator. For ANN, the inner-CV and final outer-training SMOTE seeds are defined as 1000 + 100*f_o_* + *f_i_* and 2000 + *f_o_*, respectively. For RF, the corresponding seeds are 3000 + 100*f_o_* + *f_i_* + *t* and 5000 + *f_o_*; for SVM, they are 4000 + 100*f_o_* + *f_i_* + *t* and 6000 + *f_o_*. Here, *f_o_*, *f_i_,* and *t* denote the outer-fold index, the inner-fold index, and the tuning-trial index, respectively. Neighbour selection uses standardised feature distances. For ANN and SVM, feature scaling is estimated from the corresponding training fold before oversampling. For RF, scaling is used only for the SMOTE distance calculation, while the RF classifier itself operates on the original feature scale. Oversampling is performed separately within each bridge, so interpolation does not occur between different bridges or between the prescribed north and south condition histories. Within a bridge, minority-class samples from the current training partition may be used as neighbours regardless of their date. Synthetic feature vectors are generated by linear interpolation between a minority sample and one of its *k* nearest same-bridge neighbours until class balance is reached. No imposed support-stiffness-reduction percentage is assigned to these interpolated feature vectors because such an assignment would not imply a physically simulated intermediate bridge state. Synthetic samples are used only for classifier training, whereas stiffness-reduction interval analyses and performance evaluation use original simulation samples not generated by SMOTE.

*Nested CV*: CV is a resampling strategy for model evaluation and hyperparameter tuning [52]. *k*-fold CV splits the data into *k* folds, training on *k* − 1 folds and validating on the remaining fold in a rotating manner. Averaging results estimates generalisation performance and reduces reliance on a single split. Nested CV uses an outer loop for performance evaluation and an inner loop for hyperparameter tuning, preventing information leakage by separating tuning and testing data. In this study, a 5-fold outer CV splits the data into training and test partitions, while a 3-fold inner CV selects hyperparameters within each outer training set. The five outer-fold results are then averaged to obtain the final evaluation metrics. Within the nested CV procedure, all preprocessing operations used in the final classifier evaluation, including scaling and hyperparameter optimisation, are performed only on training data. For ANN and SVM, normalisation parameters are estimated from the corresponding training fold and then applied unchanged to the inner-validation or outer-test fold, as applicable. Sensor orientations and batch sizes are treated as separate modelling configurations, and no outer-test information is used for feature selection, transfer tuning, or hyperparameter optimisation.

*Hyperparameter Tuning*: The search spaces of hyperparameters and tuning settings are summarised in Supplementary Table S2. The hyperparameters selected within the inner CV for each outer fold are reported in Supplementary Table S3: (a) ANN, (b) RF, and (c) SVM. For ANN, grid search evaluates predefined combinations of hidden-layer size, number of neurons, learning rate, activation function, and L2 regularisation within nested CV. A full nested grid search is performed separately for each sensor orientation at the reference batch size of 7. For the larger batch sizes of 15, 31, and 61, transfer tuning is applied separately for each sensor orientation: the ANN architecture selected from the corresponding 7-crossing training procedure is used as the reference configuration, while the scale-sensitive parameters, λ and learning rate, are retuned within the inner folds of the respective outer-training set. No outer-test data are used to select the reference configuration or to tune the transferred models. The inner CV uses a reduced iteration budget of 100–200 for screening, followed by retraining with 1000 iterations to ensure convergence.

For RF and SVM, random search is used to reduce the cost of exhaustive tuning. Random-search configurations are generated reproducibly using outer-fold-specific Twister seeds defined as 1000 + *f_o_* for RF and 2000 + *f_o_* for SVM, corresponding to seeds 1001–1005 and 2001–2005 across the five outer folds. The ANN workflow is initialised with Twister seed 1. RF tuning covers the minimum leaf size, maximum number of splits, and number of predictors sampled at each split. Each candidate is evaluated using 150 trees, and the selected configuration from each outer fold is retrained with 500 trees. SVM tuning covers the kernel family, Box Constraint, RBF kernel scale, and polynomial order when applicable. The best configurations are selected using the inner-CV F1 score and retrained on the full outer-training set. A nested CV is retained throughout to separate hyperparameter selection from performance evaluation.

## 3. Results

While [35] reported both bridge-level detection and support-level localisation performance within the broader framework, the present study focuses specifically on bridge-level detection and provides a more detailed analysis of classifier performance, data balancing, feature importance, stiffness-reduction intervals, and Markov-order sensitivity.

### 3.1. Model Performances

The predictive performance of the proposed framework is evaluated across different temporal batch sizes of 7, 15, 31, and 61 crossings. For each configuration, ANN, RF, and SVM classifiers are assessed using accuracy, precision, recall, and F1 score. The evaluation uses the outer loop of the nested CV, assessing performance on the corresponding outer-test folds and averaging across the five outer folds. Table 3 and Supplementary Table S5 summarise model performance for the RILA front- and rear-mounted configurations across all batch sizes and the single-crossing case. Table 3 reports F1 scores for the 5–20% and <5% classes, accuracy, and generalisation gaps. Values are mean ± standard deviation across the five outer cross-validation folds. The generalisation gap is the mean difference between outer-training and outer-test accuracy. Supplementary Table S5 reports precision and recall values.

The results show no monotonic improvement as batch size increases. Here and below, values are reported as mean ± standard deviation across the five outer folds. RF achieves the highest overall accuracy in the leading configuration at *m* = 7 (0.918 ± 0.031), whereas ANN trailing at *m* = 7 achieves the highest balanced accuracy (0.839 ± 0.051). Short batches therefore perform well in several configurations, but increasing the window further generally reduces healthy-class discrimination. This deterioration is particularly evident at *m* = 31 and *m* = 61, where healthy-class F1 and balanced accuracy decrease markedly for RF and SVM. Supplementary Table S5 provides the corresponding class-specific precision and recall values. Overall, neither sensor orientation is uniformly superior; their relative performance varies with classifier, batch size, and evaluation metric.

The generalisation gap does not decrease monotonically with batch size. For RF leading, the smallest gap occurs at *m* = 7 (0.075), increasing to 0.172 at *m* = 61. For RF trailing, it increases from 0.110 at *m* = 1 to 0.175 at *m* = 61. ANN trailing shows a smaller gap at *m* = 7 than the single-crossing case, whereas ANN leading reaches its largest gap at *m* = 61. SVM generally shows smaller gaps in the leading configuration, although the pattern again varies with batch size. These results indicate that using a longer crossing history does not inherently improve generalisation.

**Figure 6:**
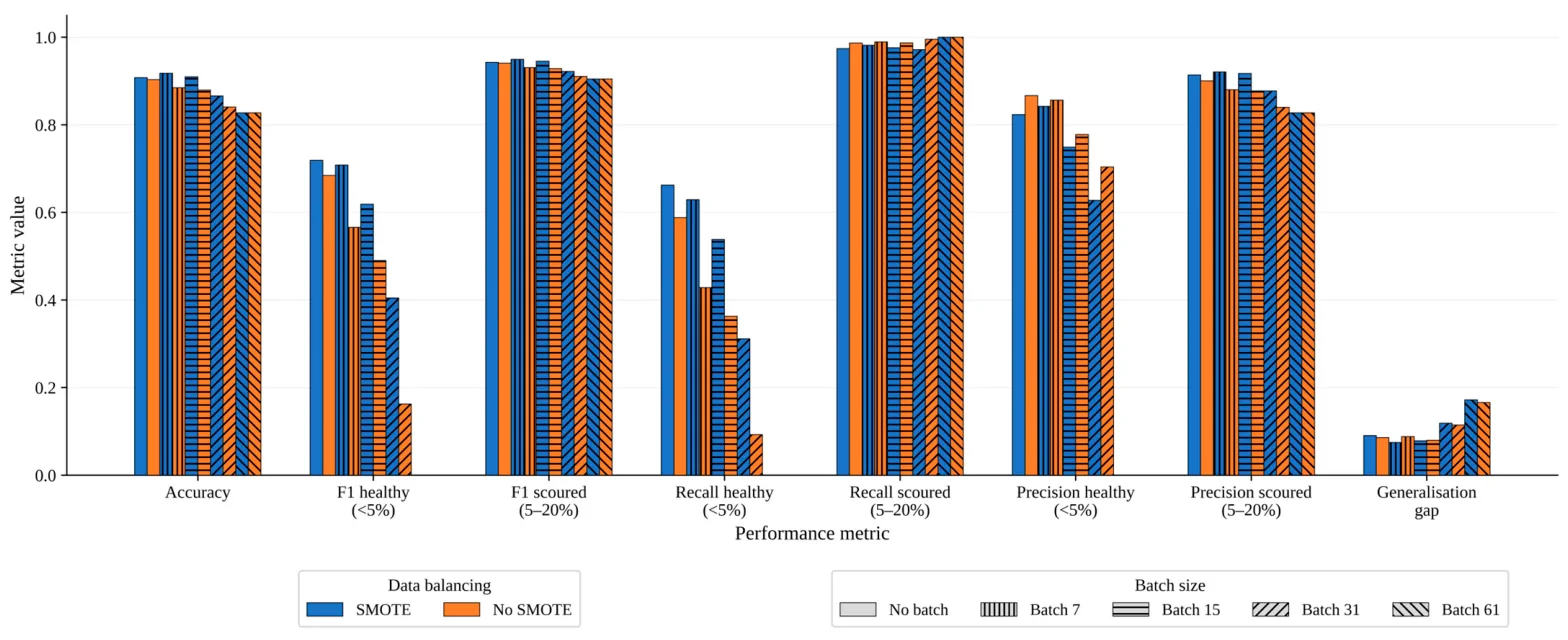
RF performance comparison with and without SMOTE: RILA leading.

**Figure 7:**
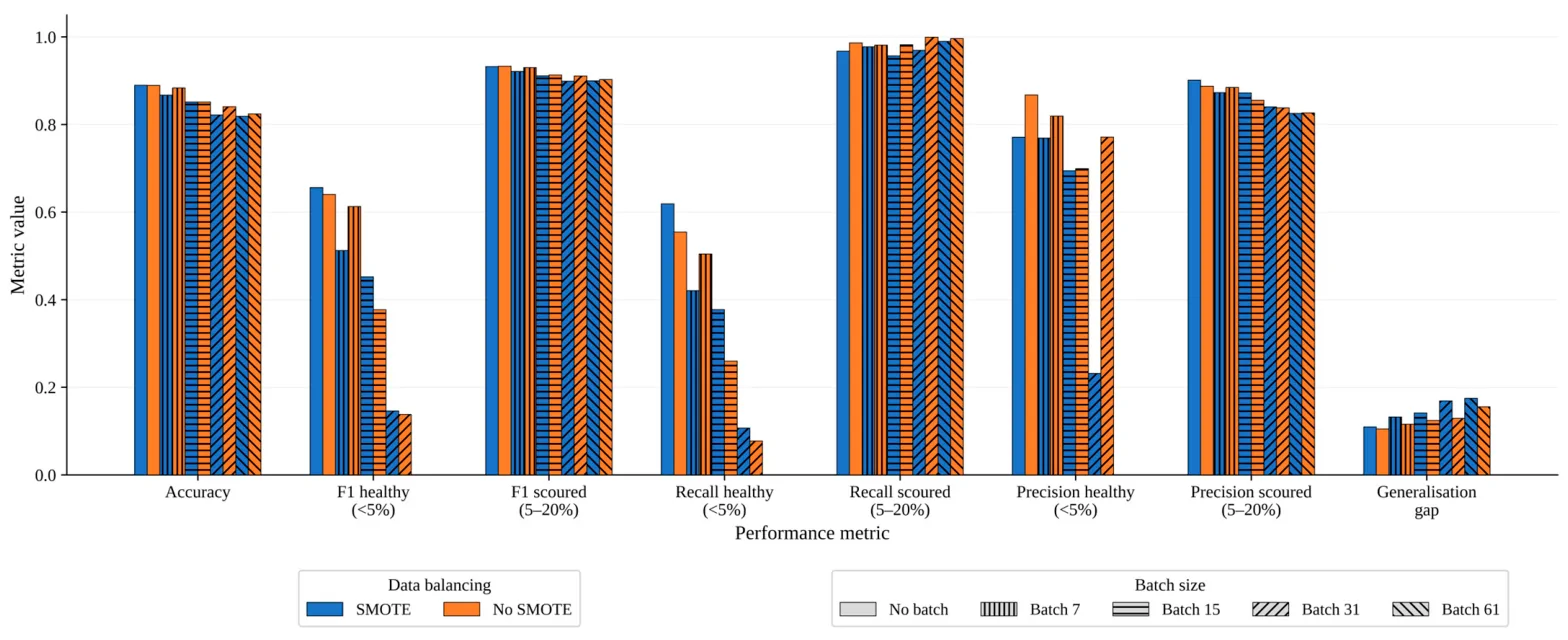
RF performance comparison with and without SMOTE: RILA trailing.

Figure 6 and Figure 7 compare RF performance with and without SMOTE for the RILA leading and trailing configurations, respectively. The comparisons use the same outer-test partitions and evaluate performance only on original held-out samples. The effect of training-fold SMOTE depends on the sensor orientation and batch size.

For the RILA leading configuration, SMOTE improves healthy-class recognition for batch sizes of 1, 7, 15, and 31 crossings. At m = 7, healthy-class F1 increases from 0.566 without SMOTE to 0.708 with SMOTE, while healthy-class recall increases from 0.429 to 0.629. Balanced accuracy consequently increases from 0.709 to 0.806. For m = 61, however, healthy-class recall remains zero with and without SMOTE, indicating that oversampling does not overcome the deterioration in healthy-class recognition at the largest batch size.

For the RILA trailing configuration, the effect of SMOTE is less consistent. At *m* = 7, healthy-class F1 decreases from 0.613 without SMOTE to 0.512 with SMOTE, while healthy-class recall decreases from 0.504 to 0.421. Correspondingly, balanced accuracy decreases from 0.743 to 0.699. Improvements are observed at *m* = 1 and *m* = 15, whereas the differences at larger batch sizes are limited or mixed. Thus, SMOTE does not uniformly improve minority-class recognition, and its benefit depends on the sensor configuration and crossing-history length. Scoured-class recall remains high in several configurations, but high scoured-class recognition alone should not be interpreted as satisfactory balanced classification performance.

**Figure 8:**
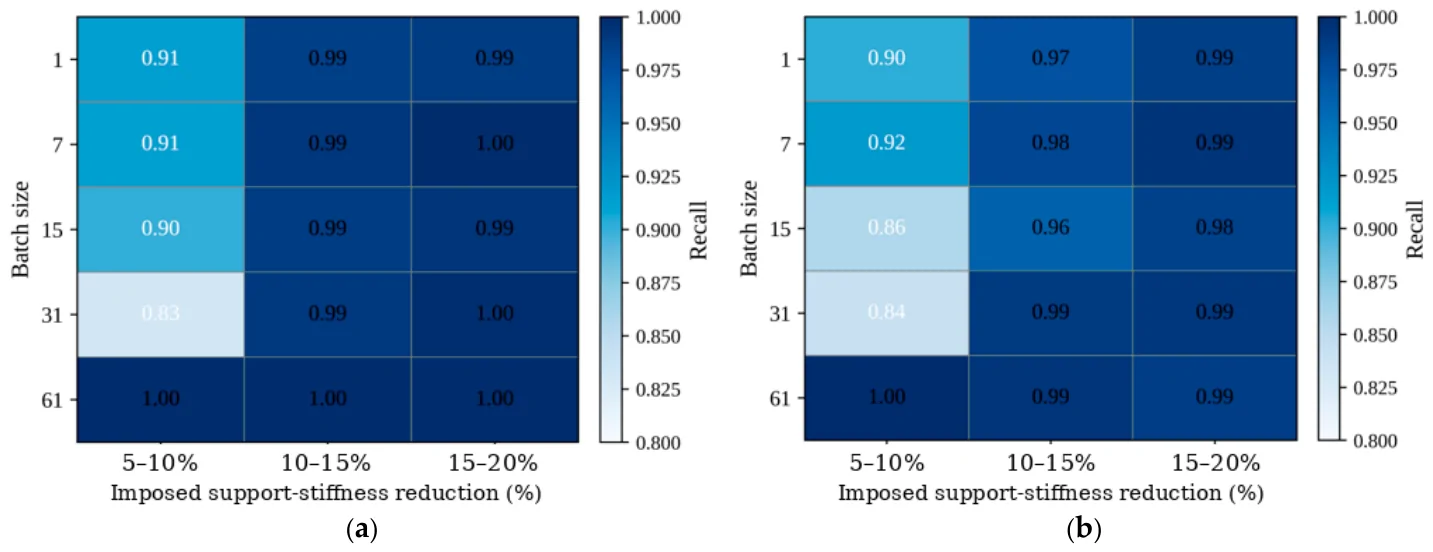
RF recall across imposed support-stiffness-reduction intervals: (**a**) RILA leading; (**b**) RILA trailing.

For RF, which achieves the highest overall accuracy, the binary detector is additionally examined across different imposed support-stiffness-reduction intervals. The classifier is not trained to predict scour severity or physical scour depth. For descriptive analysis only, scoured cases are grouped into 5–10%, 10–15%, and 15–20% stiffness-reduction intervals. Since this analysis considers only scoured cases within each interval, interval-specific accuracy is numerically equivalent to recall. Precision cannot be assigned to individual intervals because false positives arise from healthy cases, and interval-specific F1 score is therefore also not reported. Consequently, Figure 8a,b present recall only for leading and trailing configurations, respectively. Supplementary Table S8a reports the corresponding sample size n, missed-detection count (FN), recall, and 95% confidence interval for each imposed support-stiffness-reduction interval, sensor orientation, and batch size.

Figure 8 shows interval-specific recall for the original outer-test predictions from the SMOTE-trained RF models. Recall is generally lowest in the 5–10% stiffness-reduction interval. For the leading configuration (Figure 8a), recall in this interval decreases from 0.914 at *m* = 1 to 0.833 at *m* = 31. In contrast, recall for the 10–15% and 15–20% intervals remains at or above 0.986 and 0.989, respectively, over *m* = 1 to 31. For the trailing configuration (Figure 8b), recall in the 5–10% interval ranges from 0.843 to 0.919 over *m* = 1 to 31, while the corresponding minimum values for the 10–15% and 15–20% intervals are 0.962 and 0.984. The apparently high interval recall at *m* = 61 should be interpreted cautiously because the overall RF models show severely degraded healthy-class recognition at this batch size. For the complete held-out RF test population, Supplementary Table S8b reports true positives (TPs), true negatives (TNs), false positives (FPs), false negatives (FNs), and the false-alarm rate for each sensor orientation and batch size. The false-alarm rate is calculated as FP/(FP + TN). Table 3 and Supplementary Table S5 report precision and F1 score for the same held-out test population.

### 3.2. Feature-Importance Analysis

Feature importance is computed in a model-appropriate manner. For ANN and SVM, after the performance evaluation, a sensitivity analysis following Cortez and Embrechts [47] is conducted. Each feature is perturbed *L* = 100 times, and the variance of the resulting sensitivities *S_v_* is computed (Equation (19)). The relative importance is obtained by normalising *M_a_ = S_v_* via Equation (20), so importances sum to one. For RF, MATLAB’s TreeBagger out-of-bag (OOB) shuffled-feature importance is used. For each tree *t*, the OOB error *ε_t_* is measured. For each predictor *x_j_* used to split that tree, values are randomly shuffled on its OOB samples to obtain *ε_tj_*; the difference *d_tj_ = ε_tj_* − *ε_t_* is recorded. Here, *ε_t_* is the baseline OOB error of tree *t* on its own OOB samples, and *ε_tj_* is the OOB error of the same tree *t* after only the feature *x_j_* is randomly shuffled across the OOB samples, leaving all other features unchanged. Across trees, the mean *μ_j_* and standard deviation *σ_j_* of *d_tj_* are computed, and importance is reported as *μ_j_*/*σ_j_*.

For batch-based models, each original descriptor is represented by four window-level statistics and *m* crossing-specific values. Predictor-level importance values were therefore aggregated back to the 51 original descriptors before interpretation. For ANN and SVM, the normalised perturbation-based importances associated with each original descriptor were summed across its *m* + 4 window representations. For RF, the OOB permutation importances were averaged across the corresponding *m* + 4 window representations, using the values already averaged across the outer-fold models. Because these methods quantify different aspects of predictor influence and use different scales, their numerical importance values are not compared directly across classifier families; only within-model patterns and rankings are interpreted.

Supplementary Table S4 summarises the feature-importance distributions for ANN, RF, and SVM. ANN and SVM values are normalised perturbation-based relative importances, whereas RF values are OOB permutation importances averaged across the outer-fold models. For ANN, the feature-importance distribution becomes increasingly concentrated in fewer predictors for the leading configuration as batch size increases, while the trailing configuration remains more distributed. RF likewise indicates that a subset of predictors carries most of the predictive weight. However, the importance distribution flattens for larger windows, and some small negative values appear because of OOB sampling variability. SVM importance is generally distributed across the feature space, with larger local peaks in several short- and intermediate-batch cases. For the leading *m* = 61 SVM model, predictor perturbations produced zero variance in the model response, so normalised relative importance could not be estimated for that configuration. Overall, the classifiers use the expanded input space differently, supporting retention of the common 51-descriptor space for a consistent cross-model comparison. Because descriptors and their repeated window representations can be correlated, importance may be distributed or diluted among related predictors. Individual importance values are therefore interpreted cautiously, and physical conclusions are based primarily on the feature-family and grouped Markov-order ablation analyses rather than on isolated predictor rankings.

### 3.3. Markov-Order Sensitivity Analysis

Type B (Markov) features are excluded by removing early (1–5) and late (35–39) Markov orders of RF models. For each Markov-order exclusion, the RF model was independently retuned and retrained using the same leakage-free nested-CV partitions as the full model, and balanced accuracy was evaluated on the corresponding outer-test folds. Cohen’s *d* is calculated for each order and summarised as the group median to characterise class separation. This parameter clarifies where discriminative information resides. Early orders represent the initial part of the estimated impulse response, whereas later orders represent progressively delayed response information. Their relative influence may therefore reflect differences in how the front- and rear-mounted configurations encode the train–track–bridge response. Cohen’s *d* effect size [53] is calculated as seen in Equation (28):

(28) $$d_{k}=\frac{\mu_{S,k}-\mu_{H,k}}{\sqrt{\frac{\left(n_{S}-1\right)s_{S,k}^{2}+\left(n_{H}-1\right)s_{H,k}^{2}}{n_{S}+n_{H}-2}}}$$

where *μ_S,k_* and *μ_H,k_* are the mean values of Markov-order feature *k* for the scoured and healthy classes, respectively; *s*^2^*_S,k_* and *s*^2^*_H,k_* are the corresponding sample variances; and *n_S_* and *n_H_* are the respective class sample sizes. The denominator is the pooled standard deviation of feature *k*.

The RF Markov-removal experiments show only small, inconsistent changes in balanced accuracy after excluding either the early g1–g5 or late g35–*g39* orders. The reduced models were independently retuned and retrained using the same leakage-free nested-CV partitions as the full models. Neither order group produces a consistent performance reduction across batch sizes and sensor orientations. Therefore, the larger univariate effect sizes observed for the early orders in the leading configuration should not be interpreted as evidence that these orders are individually essential to RF classification. Instead, the RF models appear to distribute predictive information across multiple correlated or partially redundant descriptors. The *m* = 61 configurations are not used to infer Markov-order importance because their strongly degraded healthy-class recognition limits meaningful interpretation of feature-removal effects.

### 3.4. Feature-Family Ablation Analysis

To assess the relative contribution of the two descriptor families, an RF feature-family ablation was performed for the reference batch size m = 7 using the same outer-fold crossing partitions, chronological blocking, and window-exclusion rules as the primary RF classifier evaluation in Table 3. Four input configurations were compared: DWT-only, Markov-only, the combined 51-descriptor set, and the compact NSGA-II subset. Each feature configuration was independently tuned within the corresponding outer-training partitions through inner cross-validation, with SMOTE applied only to training data. All configurations were evaluated using identical held-out observations within each outer fold. Table 4 summarises the RF feature-family ablation results at *m* = 7. Values are balanced accuracy, reported as mean ± standard deviation across the five outer folds.

The DWT descriptors provide substantially stronger standalone discrimination than the Markov descriptors for both sensor orientations. For the leading configuration, DWT-only achieves a balanced accuracy of 0.828 ± 0.076, compared with 0.518 ± 0.030 for Markov-only. The combined configuration achieves a similar balanced accuracy of 0.832 ± 0.059, while the compact NSGA-II subset achieves 0.725 ± 0.057. For the trailing configuration, DWT-only achieves the highest balanced accuracy of 0.827 ± 0.005, followed by the compact NSGA-II subset (0.734 ± 0.095), combined (0.704 ± 0.074), and Markov-only (0.500 ± 0.030). These results indicate that the Markov descriptors have limited standalone discriminatory ability at this batch size. Their inclusion in the combined feature set produces performance comparable to DWT-only for the leading configuration but does not improve balanced accuracy for the trailing configuration. The combined configuration was independently tuned from the primary RF model reported in Table 3. Therefore, despite using identical outer-test partitions, the two evaluations are not expected to yield identical performance values.

### 3.5. Sensitivity to Alternative Condition History

To examine whether the classifier performance depends strongly on the prescribed temporal evolution of the simulated condition histories, an additional sensitivity test was performed using an abrupt healthy-to-scoured transition rather than the baseline gradual chronology. Relative to the baseline history, balanced accuracy changed by approximately +0.25 percentage points for the leading configuration and −0.56 percentage points for the trailing configuration. These small changes indicate that the main classification findings are essentially insensitive to the tested alternative temporal history.

## 4. Discussion

This study provides a detailed computational investigation of the bridge-level detection component of the broader drive-by scour monitoring framework reported in [35]. The analysis focuses on the behaviour of ANN, RF, and SVM classifiers using DWT- and ARX-derived Markov descriptors, with particular attention to data balancing, feature importance, repeated-crossing decisions, sensor orientation, and Markov-order sensitivity.

*Differences in performance across ML algorithms*: All three classifiers achieve strong scoured-class performance in several configurations, although healthy-class recognition deteriorates at larger batch sizes. ANN generally maintains high scoured-class precision, while healthy-class performance varies more. RF provides the strongest overall performance across the evaluated metrics. For the leading configuration, RF at *m* = 7 improves accuracy and reduces the generalisation gap relative to the single-crossing case. However, this pattern is not observed uniformly across sensor orientations or larger batch sizes. SVM achieves competitive accuracy, but its performance also deteriorates for the healthy class at larger batch sizes. Overall, RF provides the strongest performance within the adopted dataset and feature space, while ANN and SVM remain competitive in several configurations.

The stronger RF performance may be attributed to the input feature set, which combines DWT-based descriptors that capture profile shape and multi-resolution signal content with impulse-response parameters. RF is well suited to this feature space because it can model nonlinear interactions among scour-sensitive descriptors. Each tree can learn different threshold-based relations between predictors and class labels. Bagging and random predictor selection reduce the ensemble’s variance. This allows RF to use informative predictors and reduce sensitivity to redundant or weak predictors. ANN and SVM can also capture nonlinear patterns, but their performance is more sensitive to training settings, scaling, and the distribution of the selected feature space.

*Effect of field data*: Field-measured profiles are expected to perform worse because measurement noise, sensor drift, track defects, environmental variability, train operating conditions, and VBI calibration uncertainty may increase the mismatch between synthetic and measured data. The 0.5% RMS Gaussian-noise case used in the baseline simulations represents a nominal measurement-noise condition and should not be interpreted as evidence of field robustness. In addition, the baseline dataset retains the same bridge-specific rail-irregularity profile across crossings, so it does not capture sensitivity to temporal changes in rail condition. A further practical limitation of the ARX-based descriptors is the requirement for a reference estimate of rail irregularity in field deployment.

The present simulations do not include temperature effects because the study isolates the response to imposed foundation-support-stiffness reduction within a controlled numerical framework. A meaningful temperature sensitivity analysis would require a calibrated thermo-mechanical representation of the bridge–track system; introducing arbitrary temperature-dependent changes without such calibration would not provide a physically validated assessment. Nevertheless, temperature-dependent changes in the superstructure and track may alter measured profiles and act as a potential confounding factor in field deployment; therefore, future field-oriented validation should assess them alongside other environmental and operational variability.

The present study is limited by the absence of a systematic robustness assessment for increased measurement noise, spatial misalignment, temporal changes in rail irregularity, and uncertainty in the bridge–vehicle–track model parameters. These factors may alter the simulated track profiles and the resulting DWT and ARX/Markov descriptors, and consequently affect classifier performance. Future robustness studies should therefore quantify their influence before field deployment.

*Effect of batch size on performance*: Batch size controls the amount of recent crossing history available to the classifiers, but the results show a non-monotonic effect. Because the batch inputs are constructed from chronologically ordered synthetic histories, longer windows could in principle benefit from the persistence of similar imposed conditions across consecutive crossings; however, the alternative-history test in Section 3.5 produced only small changes in balanced accuracy. Thus, recent crossing history can provide useful context, but increasing batch length does not necessarily improve classification and may dilute information relevant to the minority healthy class.

*Model interpretability*: RFs offer greater transparency, as their tree-based structure allows feature importance to be derived directly through permutation and OOB measures, with results readily interpretable in terms of physical concepts such as Markov orders (system dynamics) and DWT bands (frequency content). In contrast, ANNs diffuse sensitivities across hidden-unit layers, and SVMs define decision boundaries through support vectors. Therefore, the decision mechanisms of ANNs and SVMs are harder to trace back to specific scour-sensitive descriptors or physical response components.

## 5. Conclusions

This study provides a detailed computational characterisation of the bridge-level detection component of a broader drive-by bridge scour monitoring framework. The analysis focuses on binary classification from simulated longitudinal track profiles using DWT- and ARX-derived Markov descriptors, together with ANN, RF, and SVM classifiers. TTB-2D v.4.4 generates synthetic drive-by longitudinal track profiles for a diverse set of trains, bridges, and multiple scour scenarios. Using scour-sensitive features derived from track profiles, binary classification algorithms detect scour. This framework combines DWT coefficients with Markov parameters for feature engineering. The impulse-response (i.e., Markov) parameters of the track profiles are obtained from ARX models. Because they are generated separately, the simulation framework allows rail irregularities to be used as exogenous inputs to the ARX models. In field applications, however, rail irregularities may not be available as a separate component, since measured track profiles include rail irregularities, bridge displacement response, noise, and other effects. In that case, impulse-response parameters could be estimated using regularised impulse-response or finite impulse-response (FIR)-type methods.

Among the evaluated ANN, RF, and SVM classifiers, RFs deliver the highest accuracy and interpretability within the synthetic framework considered. Sensor orientation affects both classifier performance and feature sensitivity, but neither the leading nor trailing configuration is consistently superior across algorithms and batch sizes. Short crossing batches improve classification in some configurations, but the benefit is not monotonic with batch length; longer windows can substantially reduce healthy-class recognition. RF retains strong scour-detection performance in several configurations with and without SMOTE, while the effect of SMOTE on healthy-class recognition varies with sensor orientation and batch size. The largest crossing batches substantially degrade healthy-class recognition regardless of oversampling.

The framework may support future monitoring practice after field validation. Since it is trained and evaluated on synthetic displacement data, the reported performance should be interpreted as a measure of numerical feasibility under simulation conditions. The classification results reflect the imposed support-stiffness reduction and do not estimate physical scour depth or severity. Some performance reduction is expected in field applications, where data from real scoured bridges remain limited. The study is also limited to bridge-level scour detection; locating the scoured pier is outside its scope. Sensitivity to increased measurement noise, spatial misalignment, temporal changes in rail irregularity, model-parameter uncertainty, and temperature effects remains to be quantified for field deployment.

## Figures and Tables

**Table 1:** Model properties of the bridges.

Bridge	No of Spans @ Span Lengths (m)	Sectional Area (m^2^)	Flexural Rigidity (kN·m^2^)	Mass per Unit Length (kg/m)	Damping Ratio (%)	Healthy Pier Spring Stiffness (kN/m)
WCM15	13.2, 5@14.0, 13.2	8.00	30.9 ∙ 10^6^	22,826	1.40	467,966
WCM15A	10.8, 7@14.0, 10.8	5.43	15.7 ∙ 10^6^	13,565	1.47	233,025
River Teign	4@23.2	2.63	30.2 ∙ 10^6^	20,622	0.50	412,777
Colonnade	3@10.83	0.13	3.65 ∙ 10^6^	997	1.65	215,375

Note: *E* = Young’s modulus, *A* = cross-sectional area, and *I* = second moment of inertia.

**Table 2:** Feature selection results.

Method	Sensor	Selected Features	MacroF1	Balanced Accuracy	MCC	F1 Score for the Scoured Class (5–20%)
RI-based & grid search	front	All	0.826	0.761	0.51	0.887
RI-based & grid search	rear	All	0.865	0.795	0.57	0.916
NSGA-II	front	*Krt*(2); *Krt*(3); *Total Energy*; *g*_33_	0.87	0.759	0.59	0.919
NSGA-II	rear	*Krt*(*A*); *Krt*(1); *Krt*(3); *L_minApp_*; *g*_10_; *g*_23_; *g*_25_	0.892	0.795	0.627	0.934

**Table 3:** Performance of ANN, RF, and SVM models using the complete 51-descriptor feature set with training-fold SMOTE.

Algorithm	Sensor	Batch Size	F1 Scoured (5–20%)	F1 Healthy (<5%)	Accuracy	Balanced Accuracy	Generalisation Gap
ANN	Leading	1	0.887 ± 0.021	0.563 ± 0.155	0.825 ± 0.023	0.765 ± 0.021	0.172
		7	0.902 ± 0.046	0.616 ± 0.099	0.848 ± 0.066	0.790 ± 0.094	0.152
		15	0.870 ± 0.072	0.472 ± 0.204	0.800 ± 0.099	0.690 ± 0.111	0.200
		31	0.871 ± 0.032	0.396 ± 0.265	0.791 ± 0.052	0.640 ± 0.150	0.209
		61	0.802 ± 0.036	0.329 ± 0.186	0.695 ± 0.061	0.588 ± 0.128	0.305
ANN	Trailing	1	0.884 ± 0.015	0.531 ± 0.163	0.818 ± 0.016	0.743 ± 0.049	0.174
		7	0.927 ± 0.028	0.697 ± 0.062	0.886 ± 0.038	0.839 ± 0.051	0.114
		15	0.924 ± 0.030	0.603 ± 0.240	0.878 ± 0.042	0.765 ± 0.112	0.122
		31	0.910 ± 0.019	0.451 ± 0.274	0.849 ± 0.032	0.670 ± 0.138	0.151
		61	0.905 ± 0.026	0.368 ± 0.276	0.839 ± 0.041	0.644 ± 0.136	0.161
RF	Leading	1	0.943 ± 0.021	0.719 ± 0.054	0.908 ± 0.030	0.818 ± 0.036	0.091
		7	0.950 ± 0.019	0.708 ± 0.150	0.918 ± 0.031	0.806 ± 0.076	0.075
		15	0.946 ± 0.019	0.619 ± 0.285	0.910 ± 0.030	0.757 ± 0.137	0.079
		31	0.922 ± 0.025	0.405 ± 0.243	0.866 ± 0.039	0.642 ± 0.095	0.119
		61	0.905 ± 0.032	0.000 ± 0.000	0.827 ± 0.055	0.500 ± 0.000	0.172
RF	Trailing	1	0.932 ± 0.025	0.656 ± 0.105	0.890 ± 0.039	0.793 ± 0.079	0.110
		7	0.921 ± 0.031	0.512 ± 0.176	0.867 ± 0.051	0.699 ± 0.085	0.132
		15	0.911 ± 0.029	0.452 ± 0.194	0.851 ± 0.044	0.667 ± 0.074	0.142
		31	0.899 ± 0.032	0.146 ± 0.213	0.822 ± 0.052	0.538 ± 0.072	0.169
		61	0.900 ± 0.035	0.000 ± 0.000	0.819 ± 0.060	0.495 ± 0.007	0.175
SVM	Leading	1	0.920 ± 0.031	0.592 ± 0.106	0.871 ± 0.045	0.746 ± 0.058	0.015
		7	0.934 ± 0.024	0.667 ± 0.049	0.892 ± 0.037	0.792 ± 0.059	0.093
		15	0.927 ± 0.028	0.587 ± 0.111	0.879 ± 0.044	0.737 ± 0.051	0.087
		31	0.912 ± 0.057	0.334 ± 0.282	0.850 ± 0.088	0.613 ± 0.120	0.023
		61	0.869 ± 0.091	0.000 ± 0.000	0.777 ± 0.134	0.469 ± 0.069	0.070
SVM	Trailing	1	0.916 ± 0.039	0.511 ± 0.106	0.859 ± 0.062	0.687 ± 0.055	0.039
		7	0.918 ± 0.030	0.583 ± 0.079	0.866 ± 0.046	0.753 ± 0.074	0.119
		15	0.914 ± 0.050	0.549 ± 0.156	0.862 ± 0.073	0.712 ± 0.074	0.114
		31	0.914 ± 0.032	0.193 ± 0.235	0.847 ± 0.056	0.561 ± 0.088	0.122
		61	0.909 ± 0.031	0.152 ± 0.189	0.838 ± 0.053	0.543 ± 0.062	0.144

**Table 4:** RF feature-family ablation at *m* = 7.

Sensor	DWT-Only	Markov-Only	Combined	NSGA-II Subset
Leading	0.828 ± 0.076	0.518 ± 0.030	0.832 ± 0.059	0.725 ± 0.057
Trailing	0.827 ± 0.005	0.500 ± 0.030	0.704 ± 0.074	0.734 ± 0.095

## Data Availability

Restrictions apply to the data used for the drive-by case study. The data are not publicly available due to restrictions related to data ownership and the project. Direct requests to access the datasets should be made to the corresponding author, who will consider them subject to the permission of the relevant data owners.

## Supplementary Materials

The following supporting information can be downloaded at: https://www.mdpi.com/article/10.3390/s26206612/s1, Table S1: Vehicle properties; Table S2: Hyperparameter search spaces for ML models; Table S3: Optimal hyperparameters for (a) ANN, (b) RF, and (c) SVM models; Table S4: Descriptor-level feature-importance analysis results after aggregation of the window-expanded predictors back to the 51 original descriptors; Table S5: Class-specific precision and recall; Table S6: Effective sample counts after excluding windows spanning CV fold boundaries; Table S7a: ARX model-order distribution and stability diagnostics; Table S7b: Comparison of the original/current ARX formulation and the direct-feedthrough formulation for the six configurations using the standard order 1–60 search; Table S8a: RF interval-specific recall across imposed support-stiffness-reduction intervals; Table S8b: Overall RF held-out confusion results and false-alarm rate; partition-manifest files providing bridge, chronological-block, outer-fold, and crossing assignments for both sensor orientations and all batch sizes; and Supplementary File S1: Partition manifests (Supplementary_Partition_Manifests.rar) containing ten Excel spreadsheets documenting bridge, chronological-block, outer-fold, and crossing assignments for both RILA sensor orientations and all five batch sizes (*m* = 1, 7, 15, 31, and 61); Supplementary Code S1: Compact MATLAB implementation (Supplementary_Code_ML_Evaluation.m) of the ANN, RF, and SVM evaluation procedure.
