# Supplementary material for: Leveraging Bridge Scour Detection by Multi-Model Machine Learning and Drive-By Data
Source: Sensors (Basel). 2026 Oct 19;26(20):6612. doi: 10.3390/s26206612 (PMC13709149; doi:10.3390/s26206612)
Supplement: Supplementary file 1 [file sensors-26-06612-s001.zip › Supplementary Table_S2.pdf]

Supplementary Table S2. Hyperparameter search spaces for ML Models.

**Model**	**Tuned hyperparameter**	**Search space / values**	**Fixed settings**
ANN	Hidden layer size	5, 10, 20, 30, 40, 50, 60, 70, 80	Full grid search; 270 configurations per external fold.
ANN	Number of hidden layers	1, 2	
ANN	Activation function	tanh	
ANN	L2 regularisation parameter: λ	10^{-6}, 10^{-5}, 10^{-4}	
ANN	Learning rate	0.001, 0.01, 0.05, 0.1	
ANN	Iteration limit	200 during inner tuning;<br>1000 for final training	
RF	Minimum leaf size	2, 4, 8, 16, 32, 64	Random search; 40 random trials per external fold.
RF	Maximum number of splits	Random integer between P and max{P, $\lfloor 0.5 \cdot P \cdot \log_2(N) + 0.5 \rfloor$}	P is the number of input features and N is the number of samples
RF	Number of predictors sampled at each split	Random integer between $\lfloor 0.5 \cdot \sqrt{P} \rfloor$ and min{P, $\lceil 1.5 \cdot \sqrt{P} \rceil$}	
RF	Number of trees	150 during tuning;<br>500 for the final outer-fold model	
RF	Split criterion	Gini diversity index (gdi)	Fixed
RF	In-bag fraction	1.0	Fixed
RF	Predictor selection	All splits	Fixed
RF	Merge leaves	off	Fixed
SVM	Kernel function	linear, RBF, polynomial	Random search;<br>60 random trials per external fold
SVM	Box constraint	Log-uniform sampling<br>from 10^{-3} to 10^{3}	
SVM	RBF kernel scale	Log-uniform sampling from 0.01 to 100; MATLAB heuristic kernel-scale estimation	
SVM	Polynomial order	2, 3, 4	
SVM	Solver	Sequential Minimal Optimisation	Fixed
