# Supplementary material for: Leveraging Bridge Scour Detection by Multi-Model Machine Learning and Drive-By Data
Source: Sensors (Basel). 2026 Oct 19;26(20):6612. doi: 10.3390/s26206612 (PMC13709149; doi:10.3390/s26206612)
Supplement: Supplementary file 1 [file sensors-26-06612-s001.zip › Supplementary Table_S3.pdf]

**Supplementary Table S3(a).** Optimal Hyperparameters for ANN Models.

| Sensor | Batch size | Outer fold | Hidden size | No. of hidden layers | Activation | λ |
|---|---|---|---|---|---|---|
| Leading | 1 | 1 | 30 | 1 | tanh | $1 \times 10^{-4}$ |
| | | 2 | 40 | 1 | | $1 \times 10^{-4}$ |
| | | 3 | 20 | 1 | | $1 \times 10^{-5}$ |
| | | 4 | 80 | 1 | | $1 \times 10^{-4}$ |
| | | 5 | 50 | 2 | | $1 \times 10^{-4}$ |
| Leading | 7 | 1 | 30 | 1 | | $1 \times 10^{-5}$ |
| | | 2 | 40 | 1 | | $1 \times 10^{-6}$ |
| | | 3 | 20 | 1 | | $1 \times 10^{-6}$ |
| | | 4 | 80 | 1 | | $1 \times 10^{-6}$ |
| | | 5 | 50 | 2 | | $1 \times 10^{-6}$ |
| Leading | 15 | 1 | 30 | 1 | | $1 \times 10^{-6}$ |
| | | 2 | 40 | 1 | | $1 \times 10^{-6}$ |
| | | 3 | 20 | 1 | | $1 \times 10^{-6}$ |
| | | 4 | 80 | 1 | | $1 \times 10^{-5}$ |
| | | 5 | 50 | 2 | | $1 \times 10^{-6}$ |
| Leading | 31 | 1 | 30 | 1 | | $1 \times 10^{-6}$ |
| | | 2 | 40 | 1 | | $1 \times 10^{-6}$ |
| | | 3 | 20 | 1 | | $1 \times 10^{-6}$ |
| | | 4 | 80 | 1 | | $1 \times 10^{-5}$ |
| | | 5 | 50 | 2 | | $1 \times 10^{-5}$ |
| Leading | 61 | 1 | 30 | 1 | | $1 \times 10^{-5}$ |
| | | 2 | 40 | 1 | | $1 \times 10^{-4}$ |
| | | 3 | 20 | 1 | | $1 \times 10^{-6}$ |
| | | 4 | 80 | 1 | | $1 \times 10^{-5}$ |
| | | 5 | 50 | 2 | | $1 \times 10^{-4}$ |
| Trailing | 1 | 1 | 20 | 2 | tanh | $1 \times 10^{-4}$ |
| | | 2 | 20 | 1 | | $1 \times 10^{-6}$ |
| | | 3 | 40 | 2 | | $1 \times 10^{-4}$ |
| | | 4 | 50 | 1 | | $1 \times 10^{-4}$ |
| | | 5 | 70 | 1 | | $1 \times 10^{-4}$ |
| Trailing | 7 | 1 | 20 | 2 | | $1 \times 10^{-4}$ |
| | | 2 | 20 | 1 | | $1 \times 10^{-6}$ |
| | | 3 | 40 | 2 | | $1 \times 10^{-4}$ |
| | | 4 | 50 | 1 | | $1 \times 10^{-6}$ |
| | | 5 | 70 | 1 | | $1 \times 10^{-6}$ |
| Trailing | 15 | 1 | 20 | 2 | | $1 \times 10^{-4}$ |
| | | 2 | 20 | 1 | | $1 \times 10^{-6}$ |
| | | 3 | 40 | 2 | | $1 \times 10^{-4}$ |
| | | 4 | 50 | 1 | | $1 \times 10^{-5}$ |
| | | 5 | 70 | 1 | | $1 \times 10^{-6}$ |
| Trailing | 31 | 1 | 20 | 2 | | $1 \times 10^{-6}$ |
| | | 2 | 20 | 1 | | $1 \times 10^{-6}$ |
| | | 3 | 40 | 2 | | $1 \times 10^{-5}$ |
| | | 4 | 50 | 1 | | $1 \times 10^{-5}$ |
| | | 5 | 70 | 1 | | $1 \times 10^{-5}$ |
| | 61 | 1 | 20 | 2 | | $1 \times 10^{-4}$ |

| | | | | | | |
|---|---|---|---|---|---|---|
| Trailing | | 2 | 20 | 1 | | $1 \times 10^{-6}$ |
| | | 3 | 40 | 2 | | $1 \times 10^{-4}$ |
| | | 4 | 50 | 1 | | $1 \times 10^{-5}$ |
| | | 5 | 70 | 1 | | $1 \times 10^{-6}$ |

**Supplementary Table S3(b).** Optimal Hyperparameters for RF Models.

| Sensor | Batch size | Outer fold | Minimum leaf size | Maximum number of splits | Predictors sampled per split |
|---|---|---|---|---|---|
| Leading | 1 | 1 | 2 | 1339 | 22 |
| | | 2 | 2 | 684 | 23 |
| | | 3 | 8 | 562 | 16 |
| | | 4 | 2 | 325 | 15 |
| | | 5 | 2 | 1438 | 24 |
| Leading | 7 | 1 | 32 | 2739 | 36 |
| | | 2 | 8 | 1766 | 27 |
| | | 3 | 32 | 871 | 33 |
| | | 4 | 16 | 1259 | 34 |
| | | 5 | 4 | 1323 | 27 |
| Leading | 15 | 1 | 64 | 2307 | 46 |
| | | 2 | 8 | 4648 | 47 |
| | | 3 | 32 | 1501 | 43 |
| | | 4 | 32 | 1532 | 45 |
| | | 5 | 32 | 2611 | 47 |
| Leading | 31 | 1 | 64 | 7267 | 57 |
| | | 2 | 32 | 4775 | 35 |
| | | 3 | 8 | 7470 | 60 |
| | | 4 | 32 | 8613 | 28 |
| | | 5 | 64 | 4099 | 46 |
| Leading | 61 | 1 | 4 | 11288 | 71 |
| | | 2 | 8 | 15495 | 55 |
| | | 3 | 2 | 9862 | 35 |
| | | 4 | 16 | 5817 | 32 |
| | | 5 | 8 | 14442 | 35 |
| Trailing | 1 | 1 | 2 | 698 | 24 |
| | | 2 | 2 | 733 | 17 |
| | | 3 | 4 | 1307 | 23 |
| | | 4 | 2 | 917 | 16 |
| | | 5 | 2 | 409 | 15 |
| Trailing | 7 | 1 | 2 | 2931 | 33 |
| | | 2 | 2 | 1499 | 35 |
| | | 3 | 4 | 1368 | 36 |
| | | 4 | 2 | 1669 | 33 |
| | | 5 | 2 | 3147 | 36 |
| Trailing | 15 | 1 | 64 | 4467 | 44 |
| | | 2 | 2 | 4972 | 46 |
| | | 3 | 2 | 3018 | 19 |
| | | 4 | 64 | 1139 | 40 |
| | | 5 | 2 | 5395 | 47 |

| | | | | | |
|---|---|---|---|---|---|
| Trailing | 31 | 1 | 64 | 7509 | 34 |
| | | 2 | 64 | 2800 | 26 |
| | | 3 | 4 | 9778 | 52 |
| | | 4 | 4 | 4536 | 38 |
| | | 5 | 8 | 7497 | 64 |
| Trailing | 61 | 1 | 4 | 10126 | 86 |
| | | 2 | 64 | 16899 | 42 |
| | | 3 | 16 | 8169 | 64 |
| | | 4 | 32 | 10391 | 73 |
| | | 5 | 16 | 15293 | 52 |

**Supplementary Table S3(c).** Optimal Hyperparameters for SVM Models.

| Sensor | Batch size | Outer fold | Kernel | Box constraint | Kernel scale | Polynomial order |
|---|---|---|---|---|---|---|
| Leading | 1 | 1 | RBF | 0.021284 | auto | — |
| | | 2 | RBF | 0.004876 | auto | — |
| | | 3 | RBF | 0.014828 | 5.324648 | — |
| | | 4 | RBF | 0.020944 | 4.788102 | — |
| | | 5 | RBF | 0.055612 | 2.795769 | — |
| Leading | 7 | 1 | RBF | 403.189841 | 28.031077 | — |
| | | 2 | RBF | 916.336637 | 19.240606 | — |
| | | 3 | RBF | 0.126947 | auto | — |
| | | 4 | RBF | 5.231558 | auto | — |
| | | 5 | RBF | 34.076009 | 17.053542 | — |
| Leading | 15 | 1 | RBF | 403.189841 | 28.031077 | — |
| | | 2 | RBF | 0.781810 | 37.541443 | — |
| | | 3 | RBF | 0.006403 | auto | — |
| | | 4 | RBF | 5.231558 | auto | — |
| | | 5 | RBF | 6.467588 | 28.712113 | — |
| Leading | 31 | 1 | Polynomial | 0.004306 | 0.047366 | 4 |
| | | 2 | RBF | 0.004876 | auto | — |
| | | 3 | RBF | 0.005026 | auto | — |
| | | 4 | RBF | 0.003864 | auto | — |
| | | 5 | RBF | 0.002116 | auto | — |
| Leading | 61 | 1 | Polynomial | 2.268202 | 2.056665 | 3 |
| | | 2 | RBF | 0.031779 | auto | — |
| | | 3 | RBF | 0.035737 | 2.912422 | — |
| | | 4 | RBF | 0.003864 | auto | — |
| | | 5 | RBF | 0.001227 | 0.190725 | — |
| Trailing | 1 | 1 | RBF | 0.175615 | auto | — |
| | | 2 | RBF | 0.101020 | 2.109929 | — |
| | | 3 | RBF | 0.035737 | 2.912422 | — |
| | | 4 | RBF | 0.004572 | auto | — |
| | | 5 | RBF | 0.055612 | 2.795769 | — |
| Trailing | 7 | 1 | RBF | 19.050719 | 36.670731 | — |
| | | 2 | RBF | 916.336637 | 19.240606 | — |
| | | 3 | RBF | 0.126947 | auto | — |
| | | 4 | RBF | 90.427268 | auto | — |

| | | | | | | |
|---|---|---|---|---|---|---|
| | | 5 | RBF | 34.076009 | 17.053542 | — |
| Trailing | 15 | 1 | Linear | 0.001833 | — | — |
| | | 2 | RBF | 802.581616 | auto | — |
| | | 3 | RBF | 0.126947 | auto | — |
| | | 4 | RBF | 1.941219 | 31.955019 | — |
| | | 5 | RBF | 6.467588 | 28.712113 | — |
| Trailing | 31 | 1 | Polynomial | 0.004306 | 0.047366 | 4 |
| | | 2 | RBF | 802.581616 | auto | — |
| | | 3 | Polynomial | 0.651581 | 31.058169 | 4 |
| | | 4 | RBF | 1.941219 | 31.955019 | — |
| | | 5 | RBF | 6.467588 | 28.712113 | — |
| Trailing | 61 | 1 | RBF | 0.072816 | 0.053144 | — |
| | | 2 | RBF | 0.031779 | auto | — |
| | | 3 | Linear | 0.002004 | — | — |
| | | 4 | Linear | 0.003839 | — | — |
| | | 5 | RBF | 176.186617 | 46.464332 | — |
