# Supplementary material for: Leveraging Bridge Scour Detection by Multi-Model Machine Learning and Drive-By Data
Source: Sensors (Basel). 2026 Oct 19;26(20):6612. doi: 10.3390/s26206612 (PMC13709149; doi:10.3390/s26206612)
Supplement: Supplementary file 1 [file sensors-26-06612-s001.zip › Supplementary Table_S4.pdf]

Supplementary Table S4. Descriptor-level feature-importance analysis results after aggregation of the window-expanded predictors back to the 51 original descriptors.

Algorithm	Sensor	Batch size	Range	Mean	SD	Negative descriptors
ANN	Leading	1	2.52×10^{-8}–0.218012	0.019608	0.048142	0
		7	1.55×10^{-10}–0.228985	0.019608	0.047850	0
		15	5.95×10^{-10}–0.328185	0.019608	0.063695	0
		31	1.28×10^{-8}–0.340481	0.019608	0.064521	0
		61	0.000045–0.782054	0.019608	0.111610	0
	Trailing	1	0–0.419304	0.019608	0.077730	0
		7	1.84×10^{-12}–0.358999	0.019608	0.061732	0
		15	0.000004–0.579952	0.019608	0.083472	0
		31	0.000024–0.510264	0.019608	0.072276	0
		61	0.000125–0.591892	0.019608	0.083030	0
RF	Leading	1	0.225906–0.616986	0.314896	0.116850	0
		7	0.093213–0.417158	0.148748	0.082753	0
		15	0.038238–0.290241	0.080124	0.057941	0
		31	0.027638–0.184669	0.054611	0.036120	0
		61	0.028862–0.103590	0.046237	0.021881	0
	Trailing	1	0.216475–0.597283	0.314203	0.108288	0
		7	0.139321–0.446617	0.199698	0.076564	0
		15	0.073628–0.305208	0.114086	0.049190	0
		31	0.054423–0.211336	0.078786	0.029990	0
		61	0.023861–0.098640	0.039940	0.015511	0
SVM	Leading	1	0.001203–0.162031	0.019608	0.034475	0
		7	0.001734–0.068566	0.019608	0.012656	0
		15	0.000599–0.070967	0.019608	0.018126	0
		31	0.000582–0.128116	0.019608	0.023101	0
		61	Not estimable*	–	–	–
	Trailing	1	0.002050–0.157117	0.019608	0.022894	0
		7	0.000288–0.170481	0.019608	0.033999	0
		15	0.000467–0.066578	0.019608	0.015056	0
		31	0.000143–0.411224	0.019608	0.060104	0
		61	0.000458–0.295604	0.019608	0.043961	0

* Not estimable: predictor perturbations produced zero variance in the model response, so normalised relative importance could not be computed.
