# Supplementary material for: Leveraging Bridge Scour Detection by Multi-Model Machine Learning and Drive-By Data
Source: Sensors (Basel). 2026 Oct 19;26(20):6612. doi: 10.3390/s26206612 (PMC13709149; doi:10.3390/s26206612)
Supplement: Supplementary file 1 [file sensors-26-06612-s001.zip › Supplementary Table_S5.pdf]

Supplementary Table S5. Class-specific precision and recall.

Algorithm	Sensor	Batch size	Precision scoured (5–20%)	Precision healthy (<5%)	Recall scoured (5–20%)	Recall healthy (<5%)
ANN	Leading	1	0.906 ± 0.048	0.535 ± 0.205	0.871 ± 0.030	0.660 ± 0.051
		7	0.909 ± 0.051	0.591 ± 0.167	0.896 ± 0.056	0.684 ± 0.155
		15	0.890 ± 0.061	0.446 ± 0.202	0.854 ± 0.096	0.527 ± 0.208
		31	0.888 ± 0.053	0.384 ± 0.242	0.858 ± 0.042	0.422 ± 0.284
		61	0.871 ± 0.047	0.277 ± 0.155	0.748 ± 0.067	0.427 ± 0.223
ANN	Trailing	1	0.896 ± 0.057	0.524 ± 0.205	0.876 ± 0.027	0.610 ± 0.120
		7	0.930 ± 0.046	0.691 ± 0.174	0.927 ± 0.048	0.751 ± 0.114
		15	0.921 ± 0.049	0.655 ± 0.299	0.931 ± 0.063	0.600 ± 0.244
		31	0.894 ± 0.046	0.540 ± 0.309	0.929 ± 0.038	0.410 ± 0.277
		61	0.884 ± 0.059	0.466 ± 0.292	0.933 ± 0.061	0.356 ± 0.320
RF	Leading	1	0.914 ± 0.044	0.824 ± 0.166	0.974 ± 0.013	0.662 ± 0.080
		7	0.921 ± 0.041	0.842 ± 0.183	0.982 ± 0.008	0.629 ± 0.155
		15	0.917 ± 0.033	0.749 ± 0.306	0.976 ± 0.015	0.538 ± 0.275
		31	0.878 ± 0.041	0.628 ± 0.368	0.972 ± 0.023	0.312 ± 0.204
		61	0.827 ± 0.055	0.000 ± 0.000	1.000 ± 0.000	0.000 ± 0.000
RF	Trailing	1	0.901 ± 0.053	0.771 ± 0.208	0.968 ± 0.018	0.619 ± 0.159
		7	0.873 ± 0.061	0.769 ± 0.287	0.978 ± 0.024	0.421 ± 0.163
		15	0.872 ± 0.053	0.695 ± 0.330	0.957 ± 0.045	0.378 ± 0.157
		31	0.840 ± 0.061	0.232 ± 0.348	0.970 ± 0.035	0.107 ± 0.154
		61	0.826 ± 0.056	0.000 ± 0.000	0.990 ± 0.014	0.000 ± 0.000
SVM	Leading	1	0.888 ± 0.058	0.724 ± 0.175	0.957 ± 0.018	0.536 ± 0.131
		7	0.902 ± 0.053	0.794 ± 0.184	0.970 ± 0.022	0.613 ± 0.126
		15	0.890 ± 0.056	0.752 ± 0.215	0.970 ± 0.018	0.505 ± 0.101
		31	0.867 ± 0.057	0.657 ± 0.461	0.964 ± 0.080	0.262 ± 0.244
		61	0.814 ± 0.066	0.000 ± 0.000	0.938 ± 0.138	0.000 ± 0.000
SVM	Trailing	1	0.862 ± 0.069	0.790 ± 0.144	0.978 ± 0.014	0.395 ± 0.115
		7	0.889 ± 0.070	0.735 ± 0.210	0.954 ± 0.044	0.551 ± 0.183
		15	0.889 ± 0.056	0.698 ± 0.229	0.945 ± 0.075	0.479 ± 0.162
		31	0.847 ± 0.061	0.782 ± 0.439	0.996 ± 0.007	0.125 ± 0.172
		61	0.842 ± 0.050	0.369 ± 0.374	0.988 ± 0.011	0.099 ± 0.131

Values are mean ± standard deviation across the five outer folds.
