# Supplementary material for: Leveraging Bridge Scour Detection by Multi-Model Machine Learning and Drive-By Data
Source: Sensors (Basel). 2026 Oct 19;26(20):6612. doi: 10.3390/s26206612 (PMC13709149; doi:10.3390/s26206612)
Supplement: Supplementary file 1 [file sensors-26-06612-s001.zip › Supplementary Table_S6.pdf]

Supplementary Table S6. Effective sample counts after excluding windows spanning CV fold boundaries.

Batch size (m)	Effective total samples	Scoured count (≥5%)	Scoured proportion (%)	Healthy count (<5%)	Healthy proportion (%)
1	2924	2338	79.96	586	20.04
7	2804	2254	80.39	550	19.61
15	2644	2142	81.01	502	18.99
31	2324	1918	82.53	406	17.47
61	1724	1426	82.71	298	17.29

Note: Effective total samples denote the retained evaluation samples after excluding sliding windows that span CV fold boundaries. For m = 1, each sample corresponds to one individual crossing; for m > 1, each sample corresponds to one retained sliding window whose class is defined by the final crossing in that window. Scoured count denotes samples with imposed support-stiffness reduction ≥5%, and Healthy count denotes samples with imposed support-stiffness reduction <5%. Class proportions are calculated relative to the effective total sample count for each batch size.
