# Supplementary material for: Leveraging Bridge Scour Detection by Multi-Model Machine Learning and Drive-By Data
Source: Sensors (Basel). 2026 Oct 19;26(20):6612. doi: 10.3390/s26206612 (PMC13709149; doi:10.3390/s26206612)
Supplement: Supplementary file 1 [file sensors-26-06612-s001.zip › Supplementary Table_S7.pdf]

Supplementary Table S7a. ARX model-order distribution and stability diagnostics.

Sensor	Bridge	Median order	Q1–Q3	Order range	Stable models (%)
Leading	WCM15	10	9–12	7–44	100
Leading	WCM15A	8	8–10	6–37	100
Leading	River Teign	8	7–8	6–27	100
Leading	Colonnade	16	7–24	5–30	100
Trailing	WCM15	11	9–14	7–47	99.18
Trailing	WCM15A	9	8–10	6–40	98.36
Trailing	River Teign	8	7–9	6–45	99.43*
Trailing	Colonnade	6	5–9	4–30	100

Note: For RILA-Trailing River Teign, ARX-specific diagnostics are based on the 348 simulations with successful parametric ARX identification. The remaining 383 cases were completed using the KNN-based imputation procedure and retained in the ML dataset.

Supplementary Table S7b. Comparison of the original/current ARX formulation and the direct-feedthrough formulation for the six configurations using the standard order 1–60 search.

Sensor	Bridge	Stable current (%)	Stable direct (%)	Residual-AC pass current (%)	Residual-AC pass direct (%)	Input–residual CCF pass current (%)	Input–residual CCF pass direct (%)	Median ΔAIC
Leading	WCM15	100	100	91.99	92.13	100	100	+0.001106
Leading	WCM15A	100	100	87.47	86.91	100	100	−0.006390
Leading	River Teign	100	100	73.16	72.34	100	100	−0.000289
Trailing	WCM15	99.18	98.50	90.85	90.85	100	100	+0.001437
Trailing	WCM15A	98.36	98.50	77.29	76.06	100	100	−0.005905
Trailing	River Teign	99.43	99.71	69.25	69.54	100	100	+0.000238

Notes: Current denotes the original shifted-input formulation with nk = 1; direct denotes the spatially aligned formulation with nk = 0, permitting direct feedthrough. Median ΔAIC is calculated as AIC direct − AIC current; therefore, negative values favour the direct-feedthrough formulation. Residual-AC and input–residual CCF pass rates use the approximate 99% correlation bounds adopted in the diagnostic analysis. The very small positive and negative ΔAIC values indicate no systematic AIC advantage for either formulation. The diagnostic script explicitly performs these comparisons at the same selected ARX order and does not regenerate the Markov features used by the ML models.
