# Supplementary material for: Leveraging Bridge Scour Detection by Multi-Model Machine Learning and Drive-By Data
Source: Sensors (Basel). 2026 Oct 19;26(20):6612. doi: 10.3390/s26206612 (PMC13709149; doi:10.3390/s26206612)
Supplement: Supplementary file 1 [file sensors-26-06612-s001.zip › Supplementary Table_S8.pdf]

Supplementary Table S8a. RF interval-specific recall across imposed support-stiffness-reduction intervals.

Batch size	Sensor	Interval	n	Missed detections (FN)	Recall	95% CI
1	Leading	5–10%	441	38	0.914	0.884–0.937
1		10–15%	648	9	0.986	0.974–0.993
1		15–20%	1249	14	0.989	0.981–0.993
1	Trailing	5–10%	448	44	0.902	0.871–0.926
1		10–15%	645	17	0.974	0.958–0.983
1		15–20%	1245	17	0.986	0.978–0.991
7	Leading	5–10%	413	36	0.913	0.882–0.936
7		10–15%	628	5	0.992	0.981–0.997
7		15–20%	1213	1	0.999	0.995–1.000
7	Trailing	5–10%	418	34	0.919	0.888–0.941
7		10–15%	627	12	0.981	0.967–0.989
7		15–20%	1209	7	0.994	0.988–0.997
15	Leading	5–10%	365	36	0.901	0.866–0.928
15		10–15%	612	7	0.989	0.977–0.994
15		15–20%	1165	8	0.993	0.987–0.997
15	Trailing	5–10%	370	53	0.857	0.817–0.889
15		10–15%	611	23	0.962	0.944–0.975
15		15–20%	1161	18	0.984	0.976–0.990
31	Leading	5–10%	275	46	0.833	0.784–0.872
31		10–15%	560	5	0.991	0.979–0.996
31		15–20%	1083	2	0.998	0.993–0.999
31	Trailing	5–10%	280	44	0.843	0.796–0.881
31		10–15%	557	6	0.989	0.977–0.995
31		15–20%	1081	9	0.992	0.984–0.996
61	Leading	5–10%	120	0	1.000	0.969–1.000
61		10–15%	469	0	1.000	0.992–1.000
61		15–20%	837	0	1.000	0.995–1.000
61	Trailing	5–10%	120	0	1.000	0.969–1.000
61		10–15%	471	3	0.994	0.981–0.998
61		15–20%	835	11	0.987	0.977–0.993

Note: n = number of held-out scoured test samples within the specified stiffness-reduction interval; FN = false negatives (missed scoured cases); Recall = TP/(TP + FN); TP = true positives; 95% CI = 95% confidence interval for recall.

Supplementary Table S8b. Overall RF held-out confusion results and false-alarm rate.

Batch size	Sensor	TP	FN	TN	FP	False-alarm rate
1	Leading	2277	61	377	209	0.357
1	Trailing	2260	78	342	244	0.416
7	Leading	2212	42	361	189	0.344
7	Trailing	2201	53	231	319	0.580
15	Leading	2091	51	314	188	0.375
15	Trailing	2048	94	203	299	0.596
31	Leading	1865	53	148	258	0.635
31	Trailing	1859	59	51	355	0.874
61	Leading	1426	0	0	298	1.000
61	Trailing	1412	14	0	298	1.000

Note: TP = true positives; FN = false negatives; TN = true negatives; FP = false positives; false-alarm rate = FP/(FP + TN). Values are calculated from the complete held-out RF test population for each sensor orientation and batch size.
