# Supplementary material for: Leveraging Bridge Scour Detection by Multi-Model Machine Learning and Drive-By Data
Source: Sensors (Basel). 2026 Oct 19;26(20):6612. doi: 10.3390/s26206612 (PMC13709149; doi:10.3390/s26206612)
Supplement: Supplementary file 1 [file sensors-26-06612-s001.zip › Supplementary Tables S1.pdf]

Supplementary Table S1. Vehicle Properties.

Parameter	Class 66/0 Co-Co Diesel	Eurostar (Locomotive + EC2–EC8 + Locomotive)	AVE S103 ICE3 (E_C1 + 2×E_C2367 + E_C45)
Train body mass (kg)	$72 \cdot 10^3$ – $88 \cdot 10^3$	Locomotive: $51.5 \cdot 10^3$<br>Coaches: 22,525	Coaches 1,2,3,4: $47.8 \cdot 10^3$<br>Coach 5: $41.2 \cdot 10^3$
Main body mass moment of inertia (kN·m²)	21,574	Locomotive: $1.05 \cdot 10^6$<br>Coaches: $810 \cdot 10^3$	Coach 1,2,3: $1.957 \cdot 10^6$<br>Coach 4: $1.687 \cdot 10^6$
Main body length (m)	13.34	Locomotive: 14.0;<br>Coaches: 18.7	17.375
Additional distance (m), front and back	4.00	Locomotive: 6.28	Coach 1: [4.76, 3.70]<br>Other coaches: [3.70, 3.70}
Number of bogies	2	2	2
Each bogie mass (kN)	8,004	Locomotive: $2.2 \cdot 10^3$<br>Coaches: $2.9 \cdot 10^3$	3,500
Bogie mass moment of inertia (kN·m²)	$19.9 \cdot 10^3$	Locomotive: 1900<br>Coaches: 2508	Coaches 1,2,3: 1,715<br>Coach 4: 1,372
Bogie length (m)	4.21	3.0	2.5
Number of axles	6	4	4
Each wheel mass (kg)	2498	Locomotive: 1700<br>Coaches: 1900	1800
Primary suspension stiffness (kN/m)	350	Locomotive: 2600<br>Coaches: 2000	2400
Primary suspension viscous damping (kN·s/m)	20	Locomotive: 12<br>Coaches: 12	20
Secondary suspension stiffness (kN/m)	1000	Locomotive: 3260<br>Coaches: 580	700
Secondary suspension viscous damping (kN·s/m)	150	90	40
